# How Oriented External Electric Fields Modulate Reactivity

**DOI:** 10.1002/chem.202004906

**Published:** 2021-01-21

**Authors:** Song Yu, Pascal Vermeeren, Trevor A. Hamlin, F. Matthias Bickelhaupt

**Affiliations:** ^1^ Department of Theoretical Chemistry Amsterdam Institute of Molecular and Life Sciences (AIMMS) Amsterdam Center for Multiscale Modeling (ACMM) Vrije Universiteit Amsterdam De Boelelaan 1083 1081 HV Amsterdam The Netherlands; ^2^ Institute for Molecules and Materials (IMM) Radboud University Heyendaalseweg 135 6525 AJ Nijmegen The Netherlands

**Keywords:** activation strain model, density functional calculations, Diels–Alder reactions, oriented external electric field, reactivity

## Abstract

A judiciously oriented external electric field (OEEF) can catalyze a wide range of reactions and can even induce *endo*/*exo* stereoselectivity of cycloaddition reactions. The Diels–Alder reaction between cyclopentadiene and maleic anhydride is studied by using quantitative activation strain and Kohn–Sham molecular orbital theory to pinpoint the origin of these catalytic and stereoselective effects. Our quantitative model reveals that an OEEF along the reaction axis induces an enhanced electrostatic and orbital interaction between the reactants, which in turn lowers the reaction barrier. The stronger electrostatic interaction originates from an increased electron density difference between the reactants at the reactive center, and the enhanced orbital interaction arises from the promoted normal electron demand donor–acceptor interaction driven by the OEEF. An OEEF perpendicular to the plane of the reaction axis solely stabilizes the *exo* pathway of this reaction, whereas the *endo* pathway remains unaltered and efficiently steers the *endo*/*exo* stereoselectivity. The influence of the OEEF on the inverse electron demand Diels–Alder reaction is also investigated; unexpectedly, it inhibits the reaction, as the electric field now suppresses the critical inverse electron demand donor–acceptor interaction.

## Graphical abstract

**It's electrifying**! Kohn–Sham molecular orbital theory is employed for the first time to rationalize how a judiciously directed oriented external electric field can steer the reactivity and selectivity of a Diels–Alder reaction. These results provide a toolbox for the design of novel electric‐field‐catalyzed organic reactions.

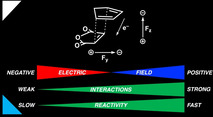

## Introduction

Recently, the study of electrostatically catalyzed non‐redox reactions has become a thriving field in chemistry.^[1]^ The reactivity, as well as selectivity, of non‐redox reactions can be manipulated by orienting the electric field in a specific direction with respect to the interacting reactants. In nature, for example, electric fields have been proposed to play a role in enzyme‐catalyzed reactions.^[2]^ In the last decade, artificially designed electric fields have also been utilized to mediate non‐redox reactions through, for example, the electrode/electrolyte interface,^[3]^ a voltage‐biased STM tip,^[4]^ and the active site under the electric field possibly created by charged functional groups^[5]^ or catalysts.^[6]^ From a theoretical point of view, a large number of studies have been dedicated to the understanding and prediction of the effect of an oriented external electric field (OEEF) on various chemical transformations^[7]^ such as C−H bond activation reactions,[^6a^, ^6b^, ^6c^, ^7a^, ^7b^, ^7c^] Diels–Alder reactions,[^5e^, ^6d^, ^7d^, ^7e^] methyl transfer reactions,^[7f]^ electrophilic aromatic substitution reactions,^[7g]^ nucleophilic substitutions of halogen‐bond complexes,^[7h]^ and oxidative addition reactions.^[7i]^

**Scheme 1:**
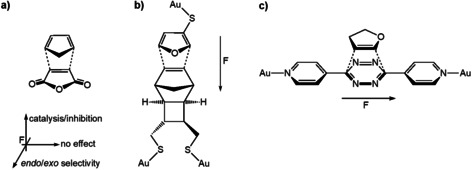
a) Theoretical predictions on the effect of the oriented external electric field (OEEF) on the Diels–Alder reaction, and experimental verifications of the OEEF b) along the reaction axis and c) aligned with the double bond of the dienophile.

The pioneering theoretical predictions made by Shaik et al. in 2010 on the effect of the OEEF on Diels–Alder (DA) reactions^[7d]^ were proven in cutting‐edge experimental studies by Coote and co‐workers six years later.^[4a]^ Shaik et al. discovered that, for the DA reaction between cyclopentadiene and maleic anhydride (Scheme 1 a), an electric field directed along the reaction axis, that is, the electric field along the forming bonds, can catalyze (positive field) or inhibit (negative field) the reaction, whereas an electric field perpendicular to the reaction axis and the bond‐forming plane will lead to an enhanced *endo* (negative field) or *exo* (positive field) selectivity. Furthermore, an electric field along the C=C double bond of maleic anhydride shows negligible effect on the reactivity or selectivity of the reaction.^[7d]^ Coote and co‐workers probed a single‐molecule DA reaction between furan and a norbornylogous bridge, which were separately tethered to a gold STM tip and gold surface, respectively (Scheme 1 b).^[4a]^ In this way, the orientation of the electric field was aligned along the reaction axis, leading to a fivefold increase in the frequency of the formation of the single‐molecule junction, observed through a so‐called “blinking” technique.^[4a]^ In addition, Hong and co‐workers confirmed, by using an electric‐field‐mediated single‐molecule reaction, that the reactivity of the studied DA reaction remains unaltered under an electric field aligned to the C=C double bond of the dienophile (Scheme 1 c).^[8]^

The molecular dipole moment has long been considered critical to understanding the effect of an OEEF on the reactivity and selectivity of a DA reaction.[^7d^, ^7e^] As the reactants and transition state of a DA reaction have distinct dipole moments along a particular direction, an OEEF is able to (de)stabilize the reactants and transition state, depending on the direction of the electric field, and hence, has an immediate effect on the activation barrier of the reaction. On the other hand, qualitative valence bond (VB) theory^[9]^ has also been utilized to understand the catalytic effect of an OEEF aligned to the reaction axis on the DA reaction. This model revealed that the charge transfer state along the reaction pathway is significantly stabilized by a positive electric field, which, as a consequence, mixes into the wavefunction at and around the transition state. This phenomenon stabilizes the transition state, and therefore lowers the activation barrier.^[7d]^ On the other hand, the OEEF‐induced *endo*/*exo* selectivity has been understood solely by the interaction between the OEEF and the molecular dipole moment in a specific stereoisomer, but has not been explained within the framework of VB theory.

**Scheme 2:**
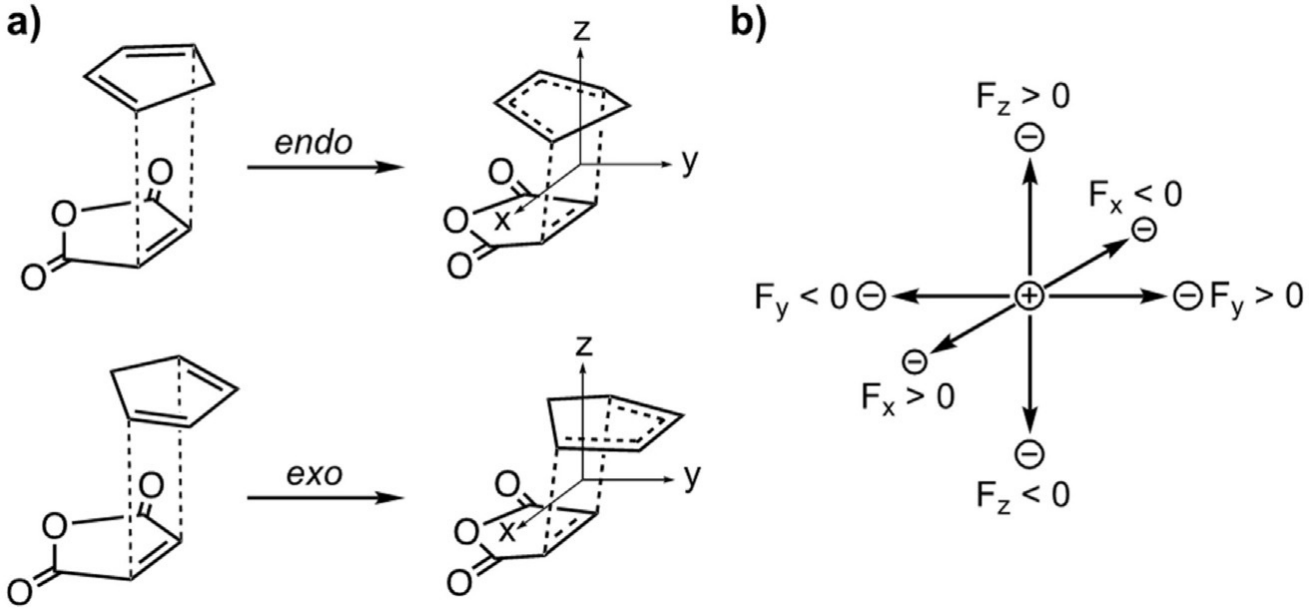
a) Schematic representation of the Diels–Alder reaction between cyclopentadiene (**Cp**) and maleic anhydride (**MA**) including the axis of the coordinate systems; b) directions of the electric fields (the electric field is defined from the positive to negative charge, as the conventional definition in physics and ADF software).

In this study, for the first time, we aim to investigate the OEEF‐mediated DA reaction within the context of Kohn–Sham molecular orbital (KS‐MO) theory. The ultimate physical factors dictating the catalytic, as well as *endo*/*exo* selective, effects of an OEEF on the Diels–Alder reaction are elucidated using quantitative KS‐MO analyses. The results obtained herein, together with the VB study of Shaik et al., effectively provide a complete framework for understanding the effects of the OEEF, and hence, will act as a toolbox for the design of novel electric‐field‐catalyzed organic reactions. To this end, we have performed a systematic computational study on OEEF‐mediated Diels–Alder reactions between cyclopentadiene (**Cp**), acting as a diene, and maleic anhydride (**MA**), acting as the dienophile (Scheme 2 a), at the BP86/TZ2P level. The activation strain model (ASM)^[10]^ of reactivity in combination with quantitative KS‐MO theory and a matching canonical energy decomposition analysis (EDA)^[11]^ have been employed to perform analyses on the Diels–Alder reactions under the OEEF along different axes. This methodology has been utilized to investigate various types of cycloaddition reactions, and has proven to be valuable for understanding the trends in reactivity.^[12]^

## Computational Methods

All calculations were performed in ADF2017^[13]^ using the BP86^[14]^ functional with the TZ2P basis set.^[15]^ The exchange‐correlation (XC) functional has been proven to be accurate in calculating the relative trends in activation and reaction energies for this reaction.[^7d^, ^12a^, ^16^] Geometries and energies were recomputed at COSMO(DCM)‐BP86/TZ2P^[17]^ to assess the effect of the solvation on the reactivity trends. Additionally, single‐point energies were computed at B3LYP/TZ2P^[18]^ and M06‐2X/TZ2P^[19]^ on the optimized BP86/TZ2P geometries to evaluate the effect of the hybrid and *meta*‐hybrid functional on the reactivity trends. Frequency calculations were performed to characterize the nature of the stationary points. Local minima present only real frequencies, whereas transition structures have one imaginary frequency. The potential energy surface (PES) was calculated using the intrinsic reaction coordinate (IRC) method,^[20]^ which follows the imaginary eigenvector of the transition structure toward the reactant and product. The resulting PES was analyzed with the aid of the PyFrag 2019 program.^[21]^ All chemical structures were illustrated using CYLview.^[22]^

Quantitative analyses of the activation barriers associated with the studied reactions were obtained by means of the activation strain model (ASM) of reactivity.^[10]^ Herein, the PES, Δ*E*(ζ), was decomposed into the strain energy, Δ*E*_strain_(ζ), and the interaction energy, Δ*E*_int_(ζ) [Eq. 1]. In this study, the reaction coordinate was projected on the length of the newly forming C⋅⋅⋅C bond, which undergoes a well‐defined change throughout the reaction and has been used in the past in analyses of similar reactions.^[12]^

(1) $$\Delta E\left(\zeta \right)=\Delta E{}_{\mathrm{strain}}\left(\zeta \right)+\Delta E{}_{\mathrm{int}}\left(\zeta \right)$$

The Δ*E*_strain_(ζ) value is associated with the rigidity as well as the structural deformation of the reactants from their equilibrium geometry to the geometry acquired along the reaction coordinate. The Δ*E*_int_(ζ) value is related to the electronic structure of the reactants and their spatial orientation, and takes the mutual interaction between the deformed reactants into account. To obtain a deeper insight into the physical mechanism behind the interaction energy, we employed canonical energy decomposition analysis (EDA).^[11]^ This analysis method decomposes the interaction energy between the two deformed reactants, within the framework of Kohn–Sham DFT, into three physically meaningful terms [Eq. 2].

(2) $$\Delta E{}_{\mathrm{int}}\left(\zeta \right)=\Delta V{}_{\mathrm{elstat}}\left(\zeta \right)+\Delta E{}_{\mathrm{Pauli}}\left(\zeta \right)+\Delta E{}_{\mathrm{oi}}\left(\zeta \right)$$

The electrostatic interaction, Δ*V*_elstat_(ζ), corresponds to the classical electrostatic interaction between the unperturbed charge distributions of the deformed reactants. The Pauli repulsion, Δ*E*_Pauli_(ζ), comprises the repulsion between closed‐shell occupied orbitals, and is, therefore, destabilizing. The orbital interaction, Δ*E*_oi_(ζ), accounts for the stabilizing orbital interactions such as electron‐pair bonding, charge transfer (interaction between the occupied orbitals of fragment A with the unoccupied orbitals of fragment B, and vice versa), and polarization (e.g., occupied–unoccupied orbital mixing on fragment A owing to the presence of fragment B, and vice versa). A detailed step‐by‐step protocol on how to perform the activation strain and energy decomposition analysis can be found in ref. [10e].

## Results and Discussion

### Definition of the oriented external electric field

The effect of an oriented external electric field (OEEF) on the reactivity and *endo*/*exo* selectivity of the Diels–Alder (DA) reactions between cyclopentadiene (**Cp**) and maleic anhydride (**MA**) is highly dependent on the direction of the field.^[7d]^ For this reason, we applied an electric field (F) individually from three distinct directions (Scheme 2 b), namely, F_*x*_, F_*y*_, and F_*z*_. These axes are defined as follows: F_*x*_ is along the C=C double bond of **MA**, F_*y*_ is perpendicular to the reaction axis, that is, perpendicular to the plane of the newly forming C−C bonds, and F_*z*_ is aligned along the reaction axis, that is, along the axis of a newly forming C−C bond. For the isolated reactants, the F_*z*_ is perpendicular to the molecular plane of **Cp** and **MA**. Shaik et al. revealed that a switch in the reaction mechanism, from a concerted to a stepwise reaction mode, will occur in solution if F_*z*_ is above 0.008 au.^[7d]^ Therefore, we limit the strength of the electric field applied in this study to ±0.008 au (1 au=514 V nm^−1^), to ensure that the reaction mechanism remains concerted for all studied electric field strengths. Note that applying an electric field will, as discussed later, make the reaction slightly asynchronous; however, this has a negligible effect on the activation barrier. In addition, this range of electric field strengths is also accessible in the laboratory.^[23]^

Table 1 displays the computed activation energies, Δ*E* ^≠^, and reaction energies, Δ*E*_rxn_, of the *endo*/*exo* Diels–Alder reactions between **Cp** and **MA** under the strongest electric fields (F=±0.008 au) along the different axes.^[24]^ An electric field along the *x* axis was found to have negligible impact on Δ*E* ^≠^ and Δ*E*_rxn_ of both the *endo* and *exo* reaction pathways. An electric field along the *y* axis, however, alters the *endo*/*exo* selectivity, namely, a negative field favors the *endo* pathway whereas a positive field goes via the *exo* pathway. Furthermore, an electric field along the *z* axis can either inhibit (negative field) or catalyze (positive field) both *endo* and *exo* reaction pathways. In the following sections, we will discuss the effects of the electric field along the various axes individually.

**Table 1:** Activation barriers (Δ*E* ^≠^, kcal mol^−1^) and reaction energies (Δ*E*_rxn_, kcal mol^−1^)^[24]^ of the *endo*/*exo* Diels–Alder reaction between **Cp** and **MA** without the electric fields (F=0) and under the electric fields (F=±0.008 au) along different axes.^[a]^

F [au]		*endo*	*endo*	*exo*	*exo*
		Δ*E* ^≠^	Δ*E*_rxn_	Δ*E* ^≠^	Δ*E*_rxn_
	0	9.6	−17.8	10.5	−18.7
*x*	−0.008	9.5	−17.8	10.4	−18.7
	0.008	9.5	−17.8	10.4	−18.7
*y*	−0.008	10.0	−18.7	12.9	−17.6
	0.008	9.9	−16.1	8.3	−19.0
*z*	−0.008	16.3	−12.5	16.7	−13.7
	0.008	0.7	−24.4	2.2	−25.0

[a] Computed at BP86/TZ2P.

### Oriented external electric field in the *z* direction

**Figure 1:**
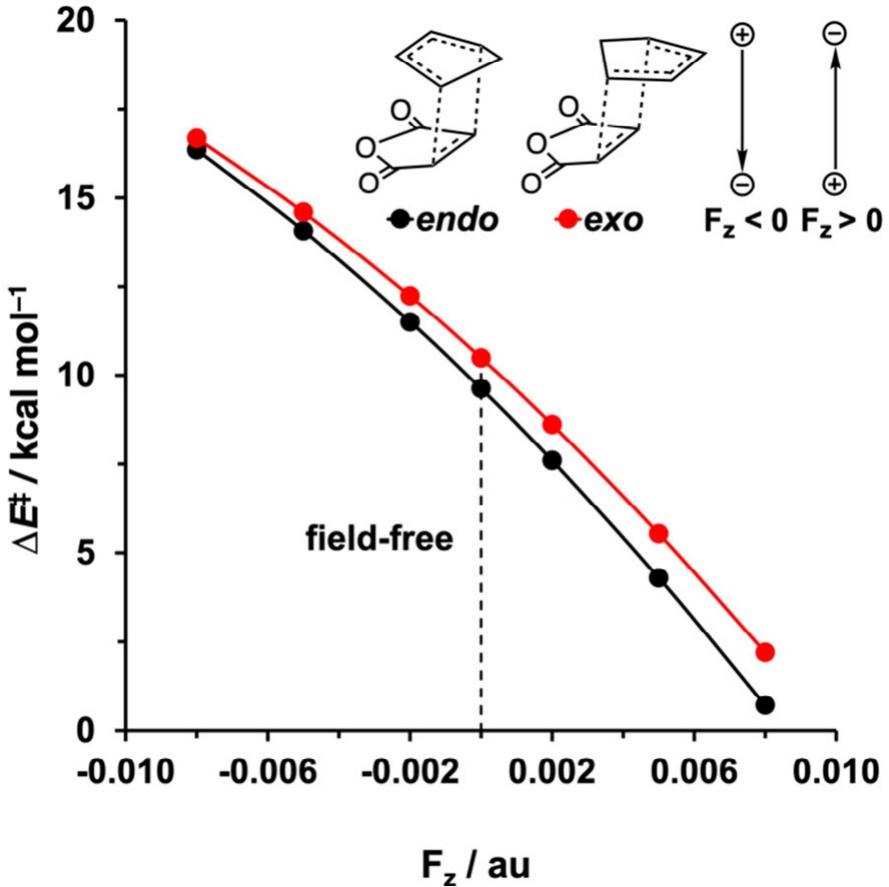
Plots of the activation energy Δ*E* ^≠^ (in kcal mol^−1^) of the *endo* and *exo* Diels–Alder reactions between **Cp** and **MA** versus the strength of the F_*z*_ (in au), computed at BP86/TZ2P.

First, we focus on the effect of the electric field in the *z* direction (F_*z*_; along the reaction axis) on the DA reactions studied herein. An electric field in the *z* direction has, as shown previously,^[7d]^ a significant catalytic (positive field) or inhibitive (negative field) effect on the DA reaction (Figure 1). A negative F_*z*_ (i.e., positive end at **Cp**, negative end at **MA**) leads to an increase in activation barrier (ΔΔ*E* ^≠^=6 kcal mol^−1^ for F_*z*_=−0.008 au), whereas a positive F_*z*_ (i.e., positive end at **MA**, negative end at **Cp**) results in a decrease in activation barrier (ΔΔ*E* ^≠^=−9 kcal mol^−1^ for F_*z*_=0.008 au), for both the *endo* and *exo* pathways. The reaction is *endo* selective for all screened F_*z*_. In line with the work of Shaik et al.,^[7d]^ the inclusion of implicit solvation in our variable OEEF calculations has no effect on reactivity trends and *endo*/*exo* selectivity (Table S1, Supporting Information).

**Figure 2:**
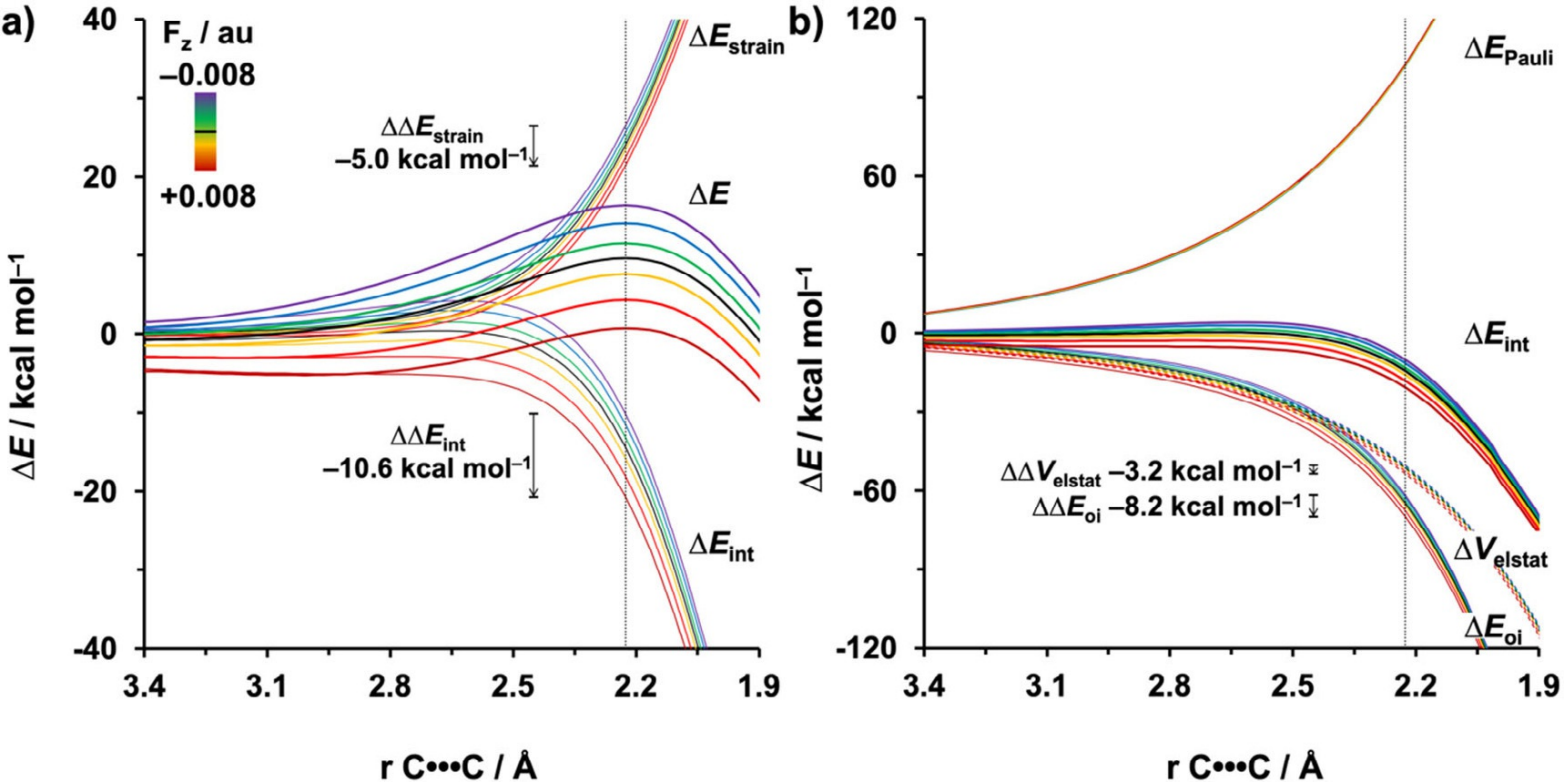
a) Activation strain and b) energy decomposition analyses of the *endo* Diels–Alder reactions between **Cp** and **MA** under F_*z*_ ranging from −0.008 to 0.008 au along the reaction coordinate projected onto the average length of the newly forming C⋅⋅⋅C bonds, computed at BP86/TZ2P. The vertical dotted line at 2.23 Å indicates the transition state.

To gain quantitative insight into the driving force leading to the catalytic or inhibitive effect of F_*z*_ on the DA reaction between **Cp** and **MA**, we turned to the activation strain model (ASM) of reactivity.^[10]^ In Figure 2 a, we focus on the activation strain diagram (ASD) of the energetically preferred *endo* pathway.^[25]^ The DA reaction is catalyzed by a positive F_*z*_ owing to both a less destabilizing Δ*E*_strain_ as well as a more stabilizing Δ*E*_int_ (Figure 2 a). Increasing F_*z*_ from −0.008 to 0.008 au leads to a Δ*E*_strain_ at the transition state that becomes 5.0 kcal mol^−1^ less destabilizing. The individual reactants undergo a deformation and reorientation over the course of the reaction, (Figure S5, Supporting Information), which results in a more favorable alignment of the dipole moment of distorted reactants with a positive F_*z*_ and hence a stabilization of these distorted reactants. As a result, the total strain energy along this reaction pathway will become less destabilizing. The stabilization of the Δ*E*_int_ at the transition state, upon increasing the F_*z*_ from −0.008 to 0.008 au, is, on the other hand, more significant, that is, ΔΔ*E*_int_=−10.6 kcal mol^−1^, indicating that the Δ*E*_int_ term is the predominant driving force leading to the catalytic or inhibitive effect of the F_*z*_ on the DA reaction.

The decisive role of Δ*E*_int_ on the reactivity prompted the analysis of the different contributors to the interaction energy Δ*E*_int_ by using a canonical energy decomposition analysis (EDA).^[11]^ The corresponding EDA results for the *endo* DA reaction between **Cp** and **MA** under the F_*z*_ ranging from −0.008 to 0.008 au are presented in Figure 2 b. We have found that the consistently more stabilizing Δ*E*_int_, as F_*z*_ is varied from −0.008 to 0.008 au, originates from both a more stabilizing Δ*V*_elstat_ and Δ*E*_oi_. The Δ*E*_Pauli_ value, on the other hand, is hardly affected by the F_*z*_, and thus, has no effect on the observed trend in reactivity.

**Figure 3:**
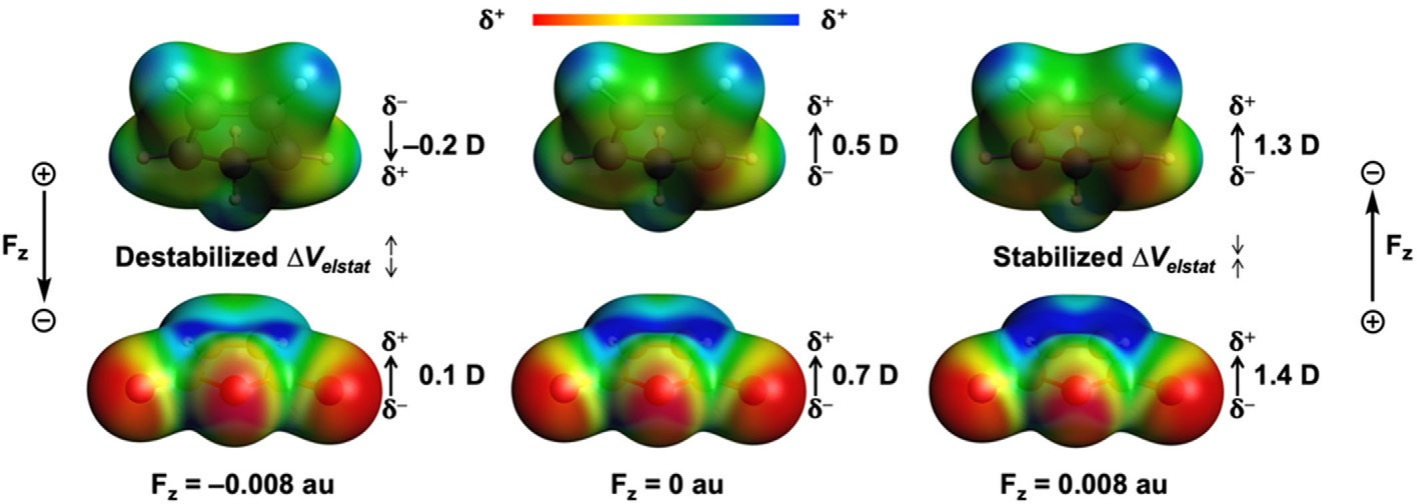
Molecular electrostatic potential maps (at 0.01 Bohr^−3^) from −0.03 (red) to 0.1 (blue) Hartree e^−1^ and dipole moments (μ_*z*_; in Debye) of isolated reactants for the *endo* Diels–Alder reactions between **Cp** and **MA** in the F_*z*_ at −0.008 au (left), 0 au (middle), and 0.008 au (right), computed at the transition‐state structures at BP86/TZ2P.

To understand the origin of the systematically more stabilizing Δ*V*_elstat_ upon going from the negative to positive F_*z*_, we analyzed the molecular electrostatic potential map (MEP) of the distorted fragments in their transition state geometry (Figure 3). From these MEPs, together with the computed dipole moment in the *z* direction (μ_*z*_), it becomes clear that the enhanced stabilization of the Δ*V*_elstat_ originates from a larger (more favorable) difference in charge density between the reactive side of the reactants going from F_*z*_=−0.008 au (left) to F_*z*_=0 au (middle) to F_*z*_=0.008 au (right) (Figure 3). For the field‐free reaction, **Cp** and **MA** have a charge separation that leads to a net negative and positive potential, respectively, on the carbon atoms involved in the formation of the new C−C bonds. These features are also reflected by their positive values of the dipole moment μ_*z*_ (**Cp**: μ_*z*_=0.5 D, **MA**: μ_*z*_=0.7 D). By applying a positive F_*z*_, the intramolecular charge separation increases and amplifies the μ_*z*_ (**Cp**: μ_*z*_=1.3 D, **MA**: μ_*z*_=1.4 D), leading to a stronger electrostatic attraction between reactants and hence a more stabilizing Δ*V*_elstat_. A negative F_*z*_, on the contrary, suppresses the μ_*z*_ (**Cp**: μ_*z*_=−0.2 D, **MA**: μ_*z*_=0.1 D), which results in a smaller difference in the charge density between reactants in the reactive regions, and thus, a less stabilizing Δ*V*_elstat_ term.

**Figure 4:**
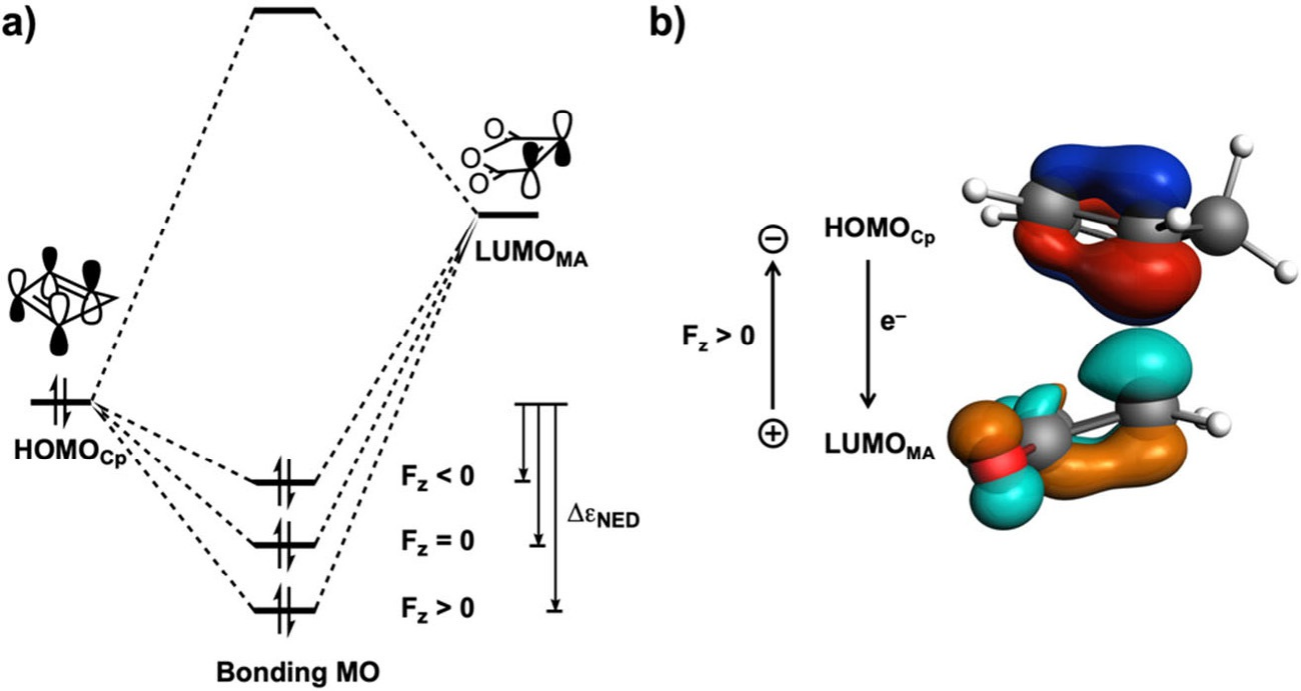
a) Schematic diagrams of the normal electron demand (NED) interaction between the HOMO_**Cp**_ and LUMO_**MA**_ for the Diels–Alder reactions between **Cp** and **MA** under different F_*z*_; b) computed HOMO_**Cp**_ and LUMO_**MA**_ (isovalue=0.03 Bohr^−3/2^) participating in the NED interaction of the field‐free reaction, including the direction of the electron flow in this interaction.

Next, Kohn–Sham molecular orbital (KS‐MO) analyses were performed to understand why Δ*E*_oi_ becomes increasingly more stabilizing from F_*z*_=−0.008 au to F_*z*_=0.008 au.[^11b^, ^26^] The normal electron demand (NED) between the HOMO_**Cp**_ and LUMO_**MA**_ is the dominant orbital interaction contributing to the Δ*E*_oi_. Analysis of the MOs reveals that the HOMO_**Cp**_ is predominantly located on the two C=C double bonds of **Cp**, whereas the LUMO_**MA**_ is centered on the C=C double bond of the five‐membered ring of **MA** (Figure 4 a). During the NED interaction, the HOMO_**Cp**_ mixes with the LUMO_**MA**_ to give a more stabilized bonding MO. The energy gain of forming this two‐center‐two‐electron interaction (i.e., orbital stabilization) relates to the energy difference between the HOMO_**Cp**_ and bonding MO (Δϵ_NED_).^[26]^ The electron density deformation associated with the NED interaction involves the flow of electrons from the HOMO_**Cp**_ to LUMO_**MA**_ and is stabilized under a positive F_*z*_ owing to the fact that the electrons move toward the positive side of the electric field (Figure 4 b), a process that goes with negative (stabilizing) work. As a result, the NED interaction is strengthened by the external electrical force, which leads to a more stabilized bonding MO, or increased Δϵ_NED_, and hence, a more stabilizing Δ*E*_oi_ (Figure 4 a). On the contrary, a negative F_*z*_ counteracts the electron flow from the HOMO_**Cp**_ to LUMO_**MA**_ because the electron is forced to move toward the negative side of the electric field, a process that results in positive (destabilizing) work. For this reason, the corresponding Δϵ_NED_ becomes smaller, quenching the NED interaction. These effects can be quantified by looking at the charge transfer from the HOMO_**Cp**_ to LUMO_**MA**_, which increases from 0.39 e to 0.50 e^−^ by changing the F_*z*_ from −0.008 to 0.008 au.

### Oriented external electric field in the *y* direction

**Figure 5:**
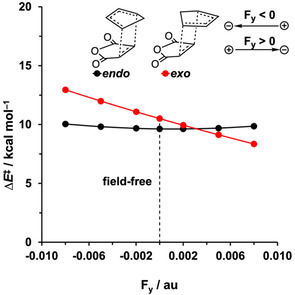
Plots of the activation energy Δ*E* ^≠^ (in kcal mol^−1^) of the *endo* and *exo* Diels–Alder reactions between **Cp** and **MA** versus the strength of the F_*y*_ (in au), computed at BP86/TZ2P.

After providing a causal model to understand how the rate of the DA reaction between **Cp** and **MA** can be tuned by an electric field along the reaction axis (F_*z*_), we examined the effect of an electric field perpendicular to the reaction axis (F_*y*_). In analogy with the work of Shaik et al.,^[7d]^ we found that F_*y*_ has a significant impact on the *endo*/*exo* selectivity of the herein studied DA reaction (Figure 5). The activation barrier of the *endo* pathway remains nearly unaffected in both a negative or positive F_*y*_, whereas the barrier for the *exo* pathway becomes systematically stabilized on going from F_*y*_=−0.008 au to F_*y*_=0.008 au. This results in a switch in the *endo*/*exo* selectivity, because an F_*y*_ of 0.003 au or higher stabilizes the *exo* pathway to such an extent that the activation barrier becomes lower than the *endo* analog.

**Figure 6:**
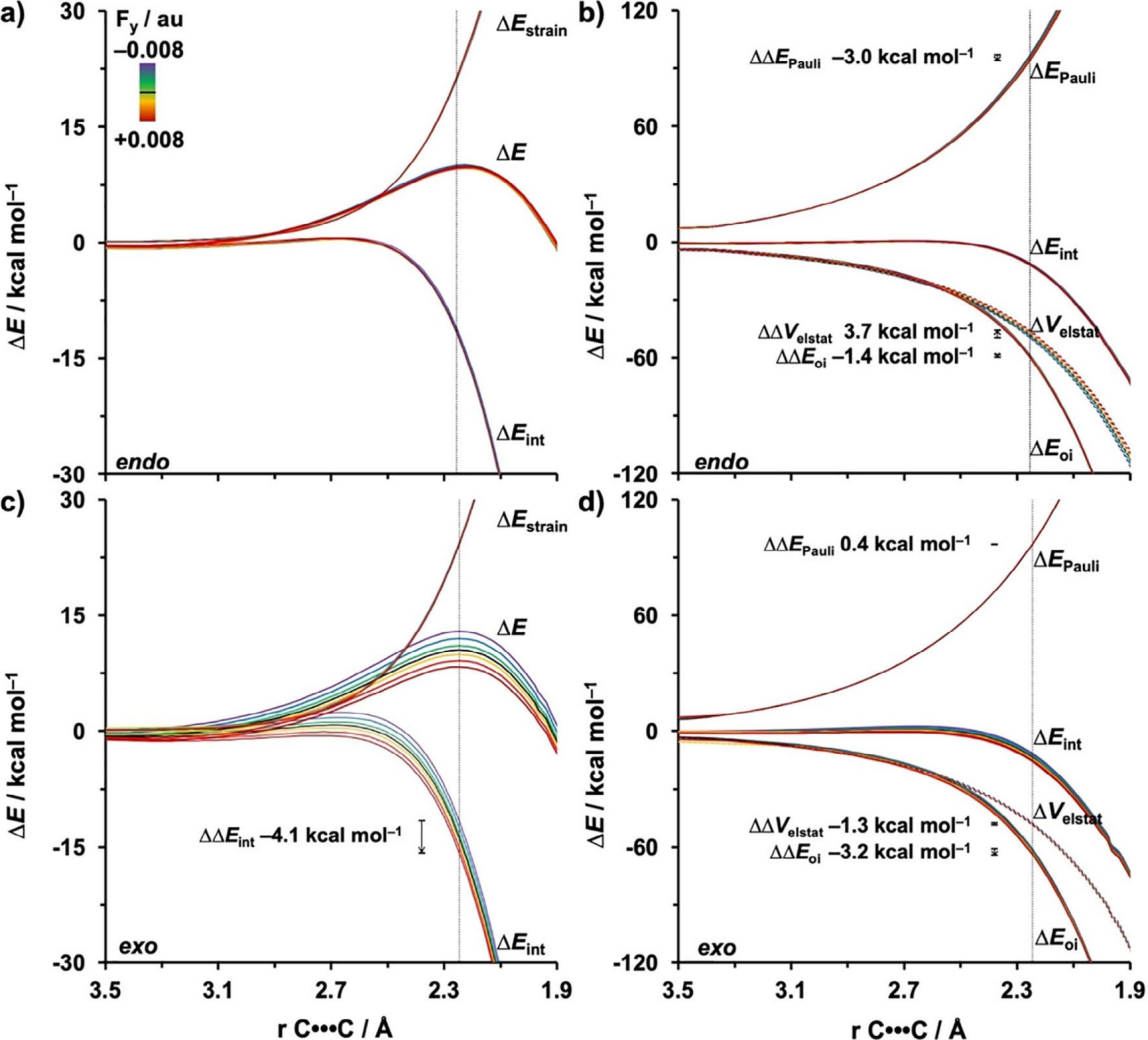
a,c) Activation strain and b,d) energy decomposition analyses of the *endo* and *exo* Diels–Alder reactions between **Cp** and **MA** in F_*y*_ ranging from −0.008 to 0.008 au, projected onto the length of newly forming C⋅⋅⋅C bonds, computed at BP86/TZ2P.

To reveal why F_*y*_ influences the *exo* activation barrier, and thus, induces a switch in the *endo*/*exo* selectivity, we again turn to the ASM. The activation barrier of the *endo* pathway remains unaltered upon applying F_*y*_ because the Δ*E*_strain_ and Δ*E*_int_ are nearly unaffected by this field (Figures 6 a). Along the *exo* pathway, the Δ*E*_int_ is increasingly more stabilizing and lowers the activation barrier as F_*y*_ increases from −0.008 to 0.008 au (Figure 6 c). Our quantitative EDA results reveal the stabilization of Δ*E*_int_ for the *exo* pathway, along this series, can be attributed to both a more stabilizing Δ*E*_oi_ and Δ*V*_elstat_ (Figure 6 d). In the next section, we will discuss why the different EDA terms along the *endo* and *exo* pathway are affected in a different manner, which ultimately explains the switch in *endo*/*exo* selectivity.

**Figure 7:**
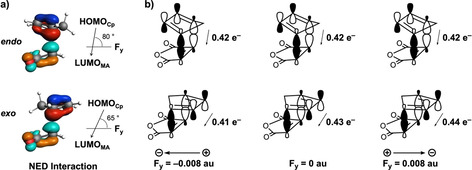
a) Computed HOMO_**Cp**_ and LUMO_**MA**_ (isovalue=0.03 Bohr^−3/2^) participating in the NED interaction for the *endo* and *exo* field‐free Diels–Alder reaction between **Cp** and **MA**; and b) schematic representation of the charge transfer in the NED interaction of the reaction under F_*y*_ at −0.008 au (left), 0 au (middle), and 0.008 au (right), computed at the transition‐state structures at BP86/TZ2P.

First, we discuss Δ*E*_oi_, which is the major contributor to the stabilization of Δ*E*_int_ for the *exo* pathway going from F_*y*_=−0.008 au to F_*y*_=0.008 au. To this end, we performed a KS‐MO analysis and identified that the NED interactions between the previously discussed HOMO_**Cp**_ and LUMO_**MA**_ are much more stabilizing than the inverse electron demand (IED) interaction HOMO_**MA**_ and LUMO_**Cp**_. The direction of the NED charge transfer with respect to the F_*y*_ determines if the electric field affects this interaction and hence catalyzes or inhibits the Diels–Alder reaction (Figure 7 a). For the *endo* pathway, both a positive and negative F_*y*_ have little effect on the electron donation capability of HOMO_**Cp**_ into LUMO_**MA**_ as F_*y*_ is nearly perpendicular (80°) to the direction of NED charge transfer between reactants (Figure 7 a). As a result, the Δ*E*_oi_, along the *endo* pathway, remains nearly unaffected upon applying an electric field in the *y* direction (Figure 7 b). In contrast, the charge transfer, and thus Δ*E*_oi_, along the *exo* pathway does become diminished (negative F_*y*_) or enhanced (positive F_*y*_) upon application of an electric field (Figure 7 b). The charge transfer accompanying the *exo* pathway is aligned more parallel to F_*y*_ (65°) (Figure 7 a), and therefore, the electron donation from the HOMO_**Cp**_ to the LUMO_**MA**_ is increased from 0.41 to 0.44 e^−^ upon varying F_*y*_ from −0.008 to 0.008 au (Figure 7 b). This amplified charge transfer stabilizes more effectively the bonding MO and leads to a larger Δϵ_NED_ (i.e., energy gap between the HOMO_**Cp**_ and bonding MO; see Figure S6, Supporting Information), and ultimately, a more favorable Δ*E*_oi_ along the *exo* pathway.

**Figure 8:**
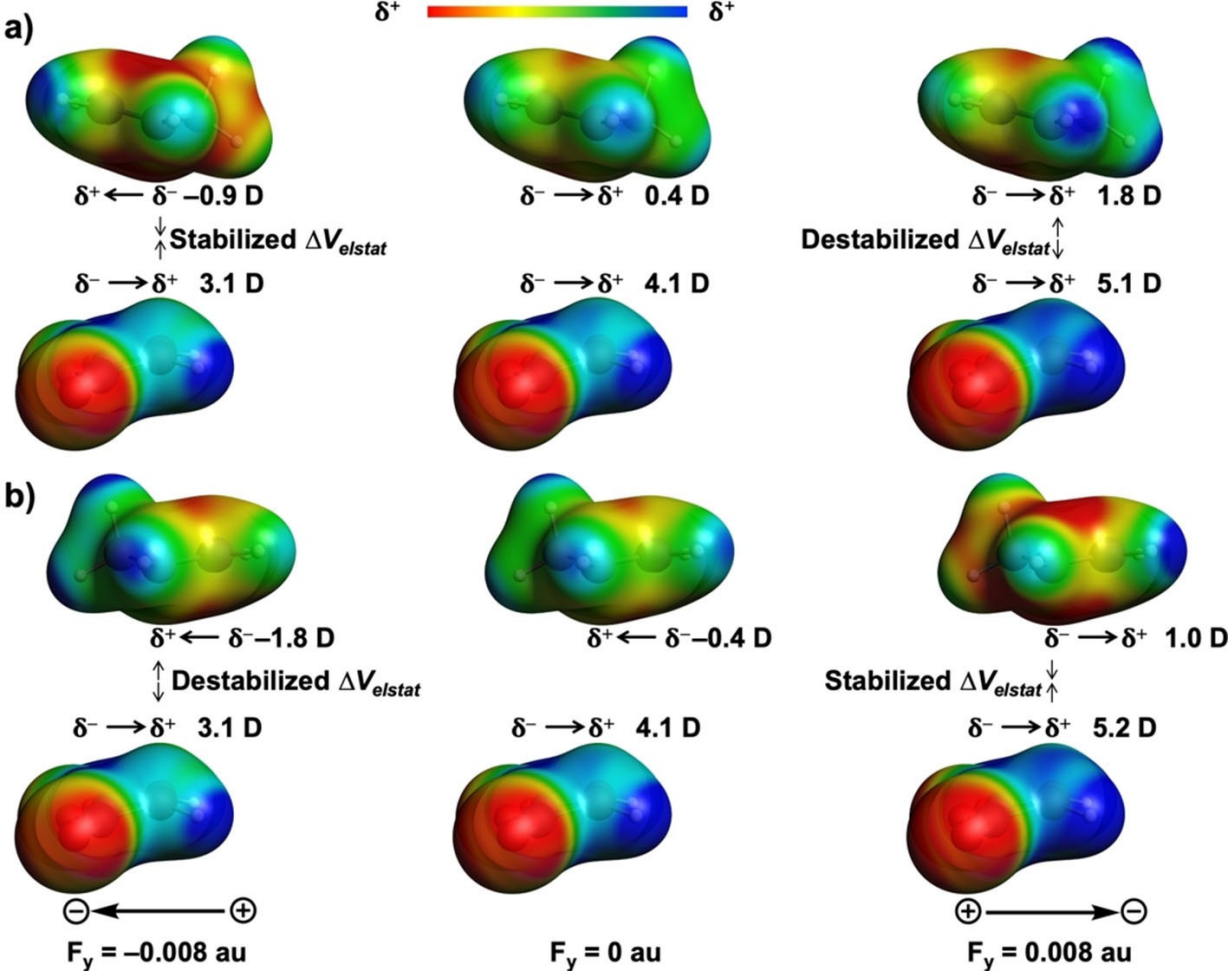
Molecular electrostatic potential maps (at 0.01 Bohr^−3^) from −0.03 (red) to 0.1 (blue) Hartree e^−1^ with dipole moments (μ_*y*_, D) of the isolated reactants of a) *endo* and b) *exo* Diels–Alder reactions between **Cp** and **MA** in the F_*y*_ at −0.008 au, 0 au, and 0.008 au, computed at the transition‐state structures at BP86/TZ2P.

Next, we analyzed Δ*V*_elstat_, which becomes increasingly less stabilizing for the *endo*, but more stabilizing for the *exo*, pathway on going from a negative to positive F_*y*_. The MEPs of the individual reactants in the geometries they obtain in the *endo* (Figure 8 a) and *exo* (Figure 8 b) transition states were generated for F_*y*_=−0.008 au (left), F_*y*_=0 au (middle), and F_*y*_=0.008 au (right). From these MEPs, together with the computed dipole moment in the *y* direction (μ_*y*_), it becomes clear that a positive F_*y*_ tends to shift the charge density toward the left (−*y* direction), whereas a negative F_*y*_ polarizes the charge density toward the right (+*y* direction). Thus, for the *endo* pathway (Figure 8 a), as F_*y*_ varies from 0 to 0.008 au, the dipole moments of the reactants become more positive (**Cp**: μ_*y*_=1.8 D; **MA**: μ_*z*_=5.1 D). The larger intramolecular charge separation leads to an enhanced electrostatic repulsion between the reactants, as both reactants have a more electron‐deficient area in the reactive center. A negative F_*y*_, on the other hand, induces an electrostatic attraction between the reactants, because the dipole moments of the reactants become smaller (**Cp**: μ_*y*_=−0.9 D; **MA**: μ_*z*_=3.1 D), resulting in an electron‐deficient (on **MA**) and accumulated (on **Cp**) area in the reactive region. For the *exo* pathway (Figure 8 b), however, the opposite behavior is observed. In this case, a positive F_*y*_ stabilizes the electrostatic attraction between the reactants, whereas a negative F_*y*_, in turn, suppresses this interaction.

The less stabilizing Δ*V*_elstat_ of the *endo* Diels–Alder reaction under a positive F_*y*_, on the other hand, is compensated by a less destabilizing Δ*E*_Pauli_, as the F_*y*_ changes the shape of the MOs that participate in the two‐center‐four‐electron orbital interaction, reducing the corresponding orbital overlap (see Figure S7, Supporting Information).^[27]^ The total interaction energy, Δ*E*_int_, along the *endo* pathway, therefore, remains nearly invariant under application of a field F_*y*_. For the *exo* pathway, on the contrary, the progressively more stabilizing Δ*V*_elstat_ and Δ*E*_oi_ lead to a more favorable Δ*E*_int_ of this reaction under a positive F_*y*_, which, in turn, lowers the activation barrier height of the *exo* pathway.

### Oriented external electric field in the *x* direction

An oriented external electric field in the *x* direction (F_*x*_) changes the Diels–Alder reaction from a concerted synchronous to a concerted slightly asynchronous reaction mode (*endo*: Δr^TS^_C⋅⋅⋅C_=0.07 Å and *exo*: Δr^TS^_C⋅⋅⋅C_=0.09 Å, where Δr^TS^_C⋅⋅⋅C_ is the difference between the newly forming C⋅⋅⋅C bonds in the TS; Figure S1, Supporting Information). This electric field, however, does not affect the reactivity or *endo*/*exo* selectivity of the DA reaction studied herein (Table S1),[^7d^, ^8^] because it is unable to either promote the charge transfer or induce a change in electrostatic interaction between the reactants, because the reactants do not have a dipole moment along the *x* axis. Shaik and co‐workers did find that an F_*x*_ induces an enantioselectivity in DA reactions between **Cp** and various asymmetric substituted ethenes such as haloethene or cyanoethene, by suppressing the formation of one of the enantiomers, which becomes highly destabilized along the pathway.^[7e]^

**Figure 9:**
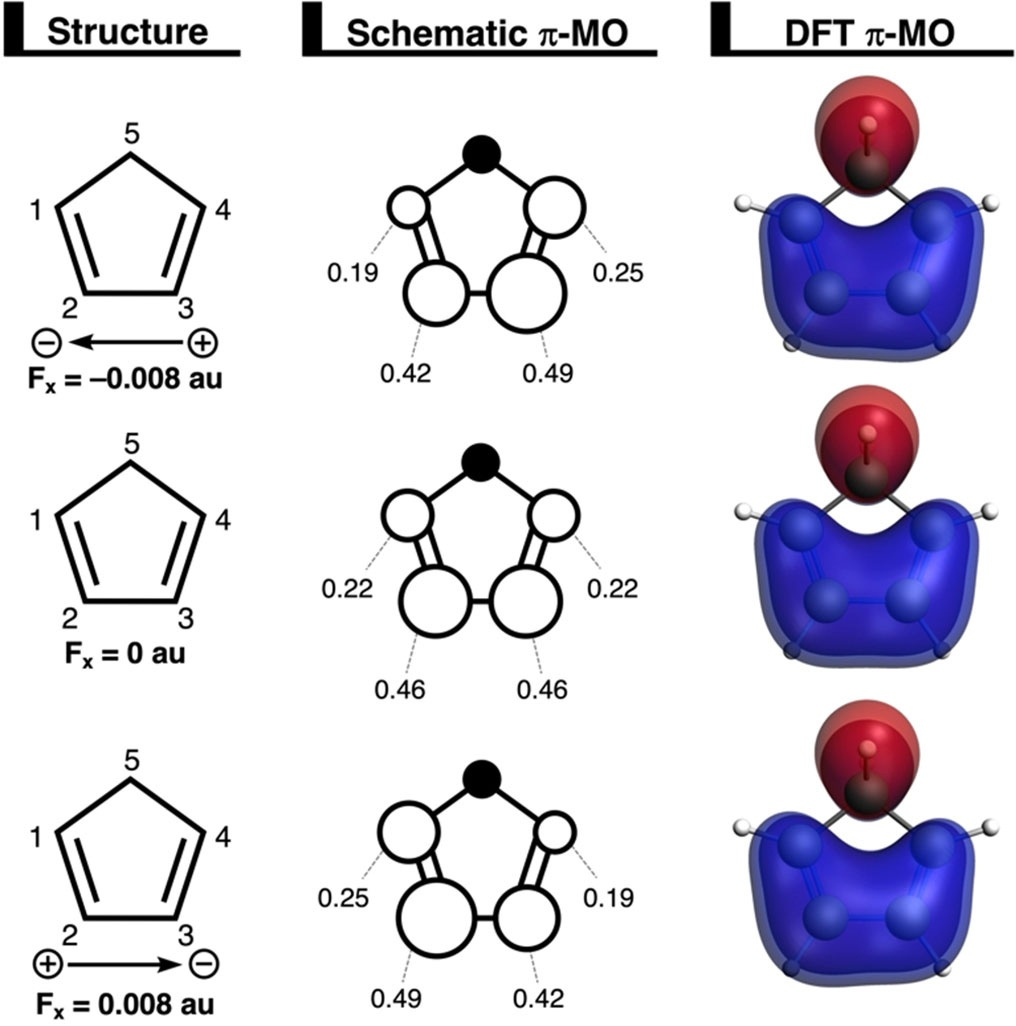
Key occupied π‐MO (isovalue=0.03 Bohr^−3/2^) computed at the equilibrium geometries of **Cp** in the F_*x*_ at −0.008 au, 0 au, and 0.008 au, in which the MO coefficients of the carbon 2p_π_ atomic orbitals, contributing to the occupied orbitals, are shown in the schematic π‐MO.

Despite the fact that F_*x*_ does not affect the reactivity or selectivity of the DA reaction, it is of interest to understand how this electric field alters the reaction mode (i.e., synchronicity) of this reaction. In our recent study, we established that the driving force behind the asynchronicity of Diels–Alder reactions is the asymmetry in the occupied orbitals of the reactants and the accompanied relief of destabilizing Pauli repulsion.^[28]^ This asymmetry introduces a bias toward the formation of one C⋅⋅⋅C bond later than the other, hence making the reaction asynchronous. Unsurprisingly, we also found this exact behavior in the DA reactions studied herein (Figure 9). In the absence of an electric field, the carbon 2p_π_ atomic orbitals (AOs) constructing the HOMO−1 of **Cp**, in which 2p_π_ AOs on the reacting C=C double bonds and the σ_C−H_ (pseudo‐π) on the methylene bridge are out‐of‐phase, are distributed symmetrically (C1_2*p*π_ and C4_2*pπ*_=0.22; C2_2*p*π_ and C3_2*pπ*_=0.46). Applying an F_*x*_ introduces an asymmetry in the HOMO−1_**Cp**_, by polarizing HOMO−1_**Cp**_ toward the positive side of the electric field. This effect of an external electric field on the spatial distribution of a molecular orbital has also been shown experimentally by using various laser‐spectroscopy techniques.^[27]^ As a result, **Cp** experiences, during the course of the Diels–Alder reaction, more Pauli repulsion with the incoming **MA** at either C1 and C2 (positive F_*x*_) or C3 and C4 (negative F_*x*_). To relieve this larger Pauli repulsion, the newly forming bond between **Cp** and **MA** at C1 (positive F_*x*_) or C4 (negative F_*x*_) remains longer than the other new bond, making the DA reaction in an electric field in the *x* direction asynchronous.

### Inverse electron demand Diels–Alder reactions

In the final section, we investigate the effect of an OEEF in the *z* direction on an inverse electron demand Diels–Alder (IED‐DA) reaction.^[29]^ The reactivity of this class of DA reactions is controlled by the IED interaction, that is, the interaction between the LUMO of diene and HOMO of dienophile.^[29]^ Based on the insight that emerged from the study of the normal electron demand DA reaction above, we expect that the F_*z*_ will have a completely opposite effect on the reactivity for the IED‐DA reaction. In other words, a positive F_*z*_ will destabilize the activation barrier by suppressing the IED interaction, and a negative F_*z*_ will now enhance the IED interaction, and therefore, lower the activation barrier.

To this end, we chose the typical IED‐DA reaction between an electron‐deficient diene, 3,6‐bis(trifluoromethyl)tetrazine (**Tz**), and cyclopentene (**Ce**) as our model (Table 2).[^16^, ^29a^, ^30^] For the first time, we show that the IED‐DA reaction between **Tz** and **Ce** is catalyzed by a negative F_*z*_ and inhibited by a positive F_*z*_. As the F_*z*_ goes from −0.008 to 0.008 au, the Δ*E* ^≠^ increases from −1.8 to 15.4 kcal mol^−1^ (Table 2). Our ASM results reveal that the increase in activation barrier is caused predominantly by the increasingly less stabilizing Δ*E*_int_ (ΔΔ*E*_int_=10.6 kcal mol^−1^), followed by a more destabilizing Δ*E*_strain_ (ΔΔ*E*_strain_=6.6 kcal mol^−1^). Next, we performed an energy decomposition analysis to pinpoint the origin of the changing Δ*E*_int_. We found that the positive F_*z*_ destabilizes the Δ*V*_elstat_ and Δ*E*_oi_, and hence, leads to a less favorable Δ*E*_int_. The less stabilizing Δ*V*_elstat_ under a more positive F_*z*_ arises from a smaller charge density difference between reactants in the reactive center (see Figure S8, Supporting Information, for MEPs). The less favorable Δ*E*_oi_ term under the positive F_*z*_, as expected, results from a weakening of the IED interaction: the positive F_*z*_ suppresses the charge transfer within the IED interaction (CT_IED_), namely, the electron donation from HOMO_**Ce**_ to LUMO_**Tz**_ (Table 2), and therefore, destabilizes the Δ*E*_oi_ term. This case, again, confirms the critical role of both the electrostatic and orbital interactions in determining the effect of electric fields on the reactivity of DA reactions.

**Table 2:** The Diels–Alder reaction between 3,6‐bis(trifluoromethyl)tetrazine (**Tz**) and cyclopentene (**Ce**) with the bonding MO of the IED interaction; and the ASM and EDA results for this reaction under the F_*z*_ at −0.008 au, 0 au, and 0.008 au, computed at the transition‐state structures at BP86/TZ2P.

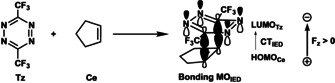							
F_*z*_ [au]	Δ*E* ^≠^	Δ*E*_strain_	Δ*E*_int_	Δ*E*_Pauli_ [kcal mol^−1^]	Δ*V*_elstat_	Δ*E*_oi_	CT_IED_ [e^−^]
−0.008	−1.8	13.6	−15.4	97.5	−55.3	−57.6	0.46
0.0	8.1	17.5	−9.4	93.8	−50.6	−52.6	0.39
0.008	15.4	20.2	−4.8	91.0	−46.9	−48.9	0.34
ΔΔ*E*	17.2	6.6	10.6	−6.5	8.4	8.7

## Conclusions

A judiciously oriented external electric field can modulate the reactivity as well as *endo*/*exo* selectivity of the Diels–Alder reaction between cyclopentadiene (**Cp**) and maleic anhydride (**MA**). A positive electric field along the forming bonds (F_*z*_>0: positive end at **MA**, negative end at **Cp**) accelerates this reaction, whereas one oriented perpendicular to the plain of the forming bonds (F_*y*_>0: positive end at the double bond of **MA**, negative end at the anhydride group of **MA**) makes the field‐free *endo‐*selective Diels–Alder reaction *exo*‐selective. These findings emerge from our quantum chemical activation strain and Kohn–Sham molecular orbital analyses based on density functional theory calculations.

The rate enhancement provoked by F_*z*_ is caused by both enhanced electrostatic and orbital interactions between the reactants. The former originates from an increased charge density difference between the reactants in the reactive region directly induced by the electric field. The positive F_*z*_ also enhances the orbital interactions by promoting the electron transfer within the normal electron demand donor–acceptor interaction between the HOMO_**Cp**_ and LUMO_**MA**_. In addition, for the *exo* pathway, a positive F_*y*_ can strengthen the orbital interactions by promoting charge transfer from HOMO_**Cp**_ to LUMO_**MA**_. The *endo* pathway, on the other hand, remains nearly unaffected, owing to a mismatch between the orientation of the reactants and the electric field. As a result, the *endo*‐selective field‐free Diels–Alder reaction becomes an *exo*‐selective Diels–Alder reaction under an adequately positive F_*y*_.

Interestingly, we have established that an F_*z*_ has an opposite effect on inverse electron demand Diels–Alder reactions, in which the most dominant orbital interaction occurs between the LUMO of the diene and HOMO of the dienophile. This orbital interaction, in contrast with the normal electron demand Diels–Alder reaction between **Cp** and **MA**, becomes strengthened by a negative F_*z*_. The results obtained herein display, for the first time, the physical factors dictating the reactivity and selectivity of Diels–Alder reactions under an external oriented electric field within the framework of Kohn–Sham molecular orbital (KS‐MO) theory, which can be applied for the understanding and design of electrostatically catalyzed reactions.

## Conflict of interest

The authors declare no conflict of interest.

## Supporting information

As a service to our authors and readers, this journal provides supporting information supplied by the authors. Such materials are peer reviewed and may be re‐organized for online delivery, but are not copy‐edited or typeset. Technical support issues arising from supporting information (other than missing files) should be addressed to the authors.

Supplementary
