# Supplementary material for: How Oriented External Electric Fields Modulate Reactivity
Source: Chemistry. 2021 Jan 21;27(18):5683–93. doi: 10.1002/chem.202004906 (PMC8049047; doi:10.1002/chem.202004906)
Supplement: Supplementary file 1 — Supplementary [file CHEM-27-5683-s001.pdf]

Chemistry–A European Journal

Supporting Information

How Oriented External Electric Fields Modulate Reactivity

Song Yu+,[a] Pascal Vermeeren+,[a] Trevor A. Hamlin,*[a] and F. Matthias Bickelhaupt*[a, b]

Contents

Table S1. Computed activation energies of the *endo*/*exo* Diels-Alder reactions between **Cp** and **MA** without electric fields (F = 0) and under the electric field (F = ±0.008 au).

Table S2. Computed activation energies (kcal mol^{-1}) of the *endo*/*exo* Diels-Alder reactions between **Cp** and **MA** without electric fields (F = 0) and under the electric field (F = ±0.008 au) at BP86/TZ2P, B3LYP/TZ2P//BP86/TZ2P, and M06-2X/TZ2P//BP86/TZ2P.

Figure S1. Optimized transition state structures of the a) *endo* and b) *exo* Diels-Alder reactions between **Cp** and **MA** in the gas phase under electric fields, computed at BP86/TZ2P.

Figure S2. Activation strain diagrams of the a) *endo* and b) *exo* Diels-Alder reactions between **Cp** and **MA** without the electric field and under the F_z of ±0.008 au. Solid lines were computed by reoptimizing the PES in the F_z, and dashed lines were obtained by single-point calculations on the PES of the field free reaction. All were computed at BP86/TZ2P in the gas phase.

Figure S3. Activation strain diagrams of the a) *endo* and b) *exo* Diels-Alder reactions between **Cp** and **MA** without the electric field and under the F_y of ±0.008 au. Solid lines were computed by reoptimizing the PES in the F_z, and dashed lines were obtained by single-point calculations on the PES of the field free reaction. All were computed at BP86/TZ2P in the gas phase.

Figure S4. a) Activation strain and b) energy decomposition analyses of the *exo* Diels-Alder reactions between **Cp** and **MA** under the F_z ranging from –0.008 to 0.008 au along the reaction coordinate projected onto the average length of the newly forming C•••C bonds, computed at BP86/TZ2P. The vertical dotted line at 2.25 Å indicates the transition state.

Figure S5. Decomposition of the ΔE_{strain} of the *endo* Diels-Alder reactions between **Cp** with **MA** under the F_z ranging from –0.008 to 0.008 au, projected onto the newly forming bond length. All results were computed at BP86/TZ2P.

Figure S6. Diagrams of the NED interactions for the *endo* (top) and *exo* (bottom) Diels-Alder reactions between **Cp** and **MA** under a F_y at –0.008, 0, and 0.008 au, computed at the transition states structures at BP86/TZ2P.

Figure S7. a) Schematic diagram for the HOMO–$1_{\mathbf{Cp}}$–HOMO–$2_{\mathbf{MA}}$ interaction of the Diels-Alder reaction between **Cp** and **MA** (*endo* as the example); b) the HOMO–$1_{\mathbf{Cp}}$|HOMO–$2_{\mathbf{MA}}$ overlaps and computed MOs of isolated reactants (isovalue = 0.06 Bohr$^{-3/2}$) for the *endo* and *exo* reactions under the F_y at –0.008 au, 0 au, and 0.008 au, where F_y is perpendicular to the reaction axis, *i.e.*, perpendicular to the plane of the newly forming C–C bonds, computed at the transition state structures at BP86/TZ2P.

Figure S8. Molecular electrostatic potential maps (at 0.01 Bohr^{-3}) from −0.03 (red) to 0.1 (blue) Hartree e^{-1} and the dipole moments (μ_z, D) of isolated reactants for the Diels-Alder reactions between **Tz** and **Ce** under the F_z at –0.008, 0, and 0.008 au, where F_z is aligned along the reaction axis, i.e., along the axis of a newly forming C–C bond, computed at transition states at BP86/TZ2P.

Table S3. Cartesian coordinates (in Å), energies (***E***, ***G***, in kcal mol^{-1}), number of imaginary frequencies ($\boldsymbol{N}_{\text{imag}}$), and the wavelength of the imaginary frequency (v, i cm^{-1}), of all stationary points, computed at BP86/TZ2P.

Table S1. Computed activation energies (kcal mol^{-1})[a] of the *endo*/*exo* Diels-Alder reactions between **Cp** and **MA** without electric fields (F = 0) and under the electric field (F = ±0.008 au).

Adduct		F / au	Spt[b]				Opt_{gas}[c]		Opt_{sol}[d]	
			$\Delta E^{‡}$	$\Delta H^{‡}$	$\Delta(-TS)^{‡}$	$\Delta G^{‡}$	$\Delta E^{‡}$	$\Delta G^{‡}$	$\Delta E^{‡}$	$\Delta G^{‡}$
endo		0	9.6	10.7	13.3	24.0	9.6	24.0	8.8	23.1
	x	–0.008	9.5	8.2	16.6	24.8	9.9	24.8	8.7	23.2
		0.008	9.5	8.2	16.6	24.8	9.9	24.8	8.7	23.2
	y	–0.008	10.0	8.8	15.2	24.0	10.0	23.9	9.9	23.8
		0.008	9.9	9.0	14.8	23.8	9.9	23.9	8.3	22.3
	z	–0.008	16.3	9.5	16.7	26.3	16.2	31.1	17.5	32.6
		0.008	0.7	–3.6	16.1	12.6	0.3	11.0	–5.3	8.2
exo		0	10.5	11.6	13.2	24.8	10.5	24.8	10.5	24.5
	x	–0.008	10.4	9.7	15.5	25.2	10.7	25.5	10.1	24.1
		0.008	10.4	9.7	15.5	25.2	10.7	25.5	10.1	24.0
	y	–0.008	12.9	11.1	16.8	27.9	12.9	26.8	14.4	28.2
		0.008	8.3	7.9	12.9	20.8	8.3	18.5	5.0	19.8
	z	–0.008	16.7	9.9	16.3	26.2	16.5	31.4	18.6	33.6
		0.008	2.2	–2.7	17.4	14.7	1.8	16.1	–3.1	10.1

[a] Computed at BP86/TZ2P. The F_x is defined along the C=C double bond of **MA**, F_y is perpendicular to the reaction axis, i.e., perpendicular to the plane of the newly forming C–C bonds, and F_z is aligned along the reaction axis, i.e., along the axis of a newly forming C–C bond. [b] Single-point calculations using non-OEEF geometries at BP86/TZ2P. [c] Optimized in the presence of the electric field in the gas phase at BP86/TZ2P. [d] Optimized in the presence of the electric field at COSMO(DCM)-BP86/TZ2P.

Table S2. Computed activation energies (kcal mol^{-1}) of the *endo*/*exo* Diels-Alder reactions between **Cp** and **MA** without electric fields (F = 0) and under the electric field (F = ±0.008 au) at BP86/TZ2P, B3LYP/TZ2P//BP86/TZ2P, and M06-2X/TZ2P//BP86/TZ2P.

Adduct		F / au	$\Delta E^{‡}$ / kcal mol^{-1}		
			BP86	B3LYP	M06-2X
endo		0	9.6	17.0	7.6
	x	–0.008	9.5	17.3	8.0
		0.008	9.5	17.3	8.0
	y	–0.008	10.0	17.3	8.0
		0.008	9.9	17.1	7.9
	z	–0.008	16.3	23.8	14.5
		0.008	0.7	7.8	–1.4
exo		0	10.5	18.5	10.0
	x	–0.008	10.4	18.8	10.3
		0.008	10.4	18.8	10.3
	y	–0.008	12.9	20.8	12.2
		0.008	8.3	16.3	8.0
	z	–0.008	16.7	24.8	16.3
		0.008	2.2	9.9	1.7

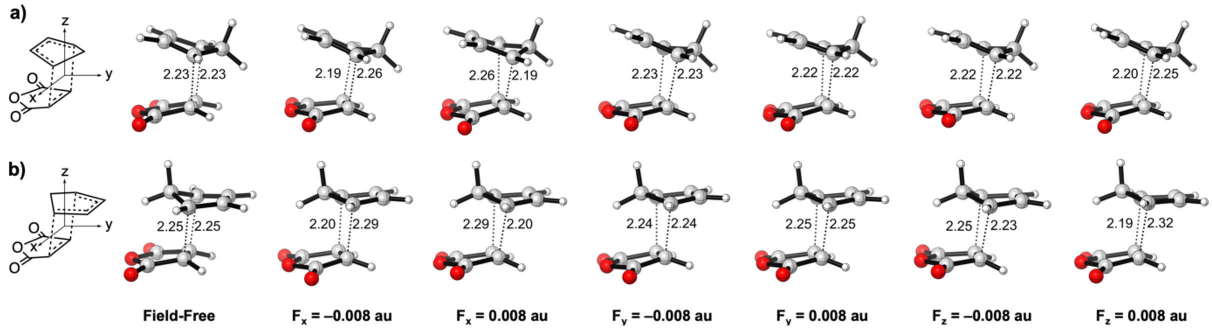

Figure S1. Optimized transition state structures of the a) *endo* and b) *exo* Diels-Alder reactions between **Cp** and **MA** in the gas phase under electric fields, computed at BP86/TZ2P.

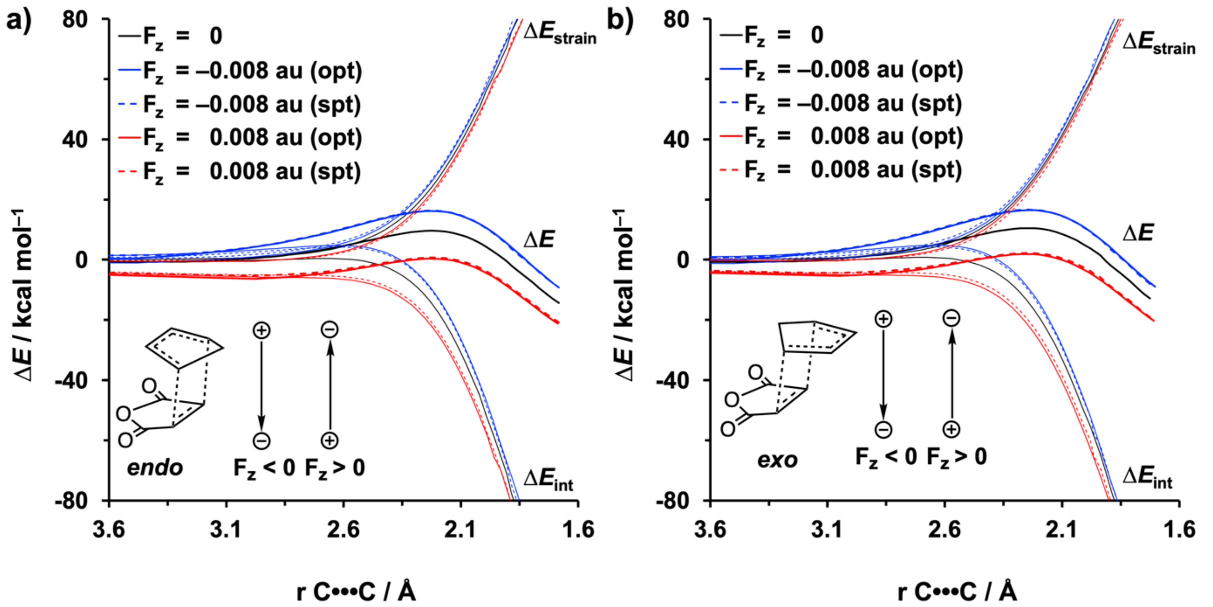

Figure S2. Activation strain diagrams of the a) *endo* and b) *exo* Diels-Alder reactions between **Cp** and **MA** without the electric field and under the F_z of ±0.008 au, where energy terms are projected on the average length of newly forming bonds. Solid lines were computed by reoptimizing the PES in the F_z, and dashed lines were obtained by single-point calculations on the PES of the field free reaction. All were computed at BP86/TZ2P in the gas phase.

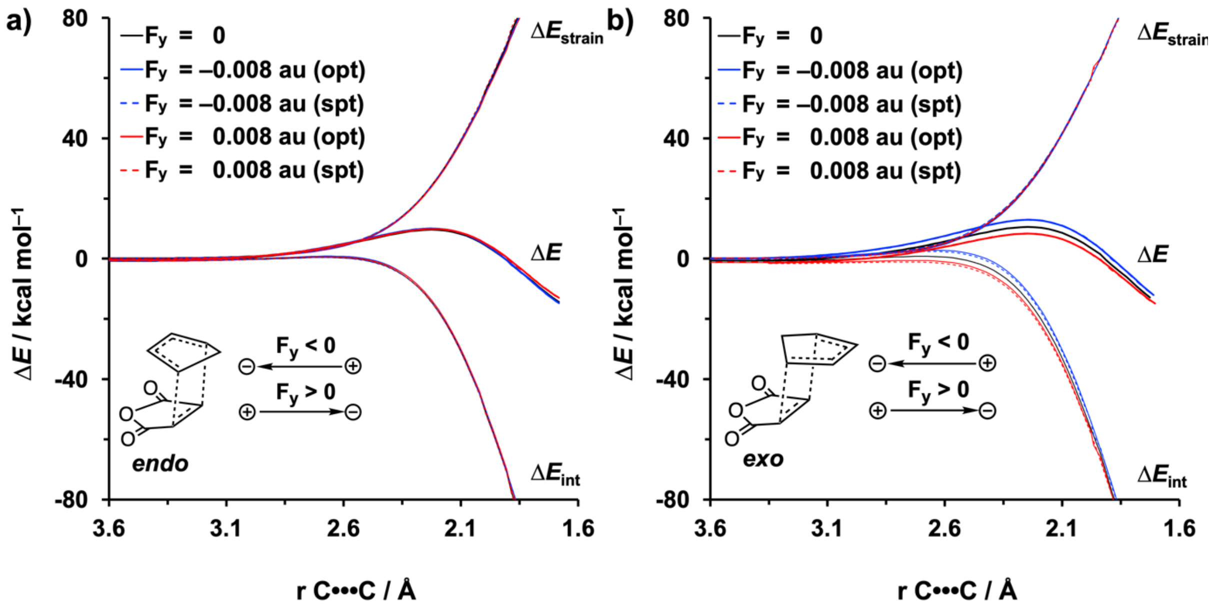

Figure S3. Activation strain diagrams of the a) *endo* and b) *exo* Diels-Alder reactions between **Cp** and **MA** without the electric field and under the F_y of ±0.008 au, where energy terms are projected on the average length of newly forming bonds. Solid lines were computed by reoptimizing the PES in the F_z, and dashed lines were obtained by single-point calculations on the PES of the field free reaction. All were computed at BP86/TZ2P in the gas phase.

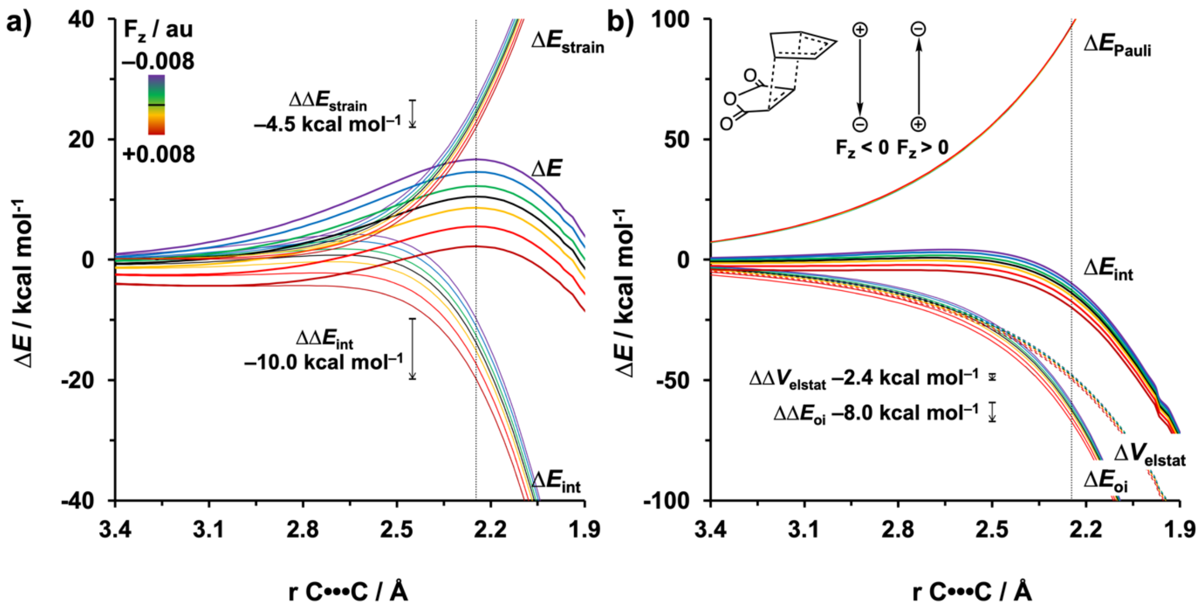

Figure S4. a) Activation strain and b) energy decomposition analyses of the *exo* Diels-Alder reactions between **Cp** and **MA** under the F_z ranging from –0.008 to 0.008 au along the reaction coordinate projected onto the average length of the newly forming C•••C bonds, computed at BP86/TZ2P. The vertical dotted line at 2.25 Å indicates the transition state.

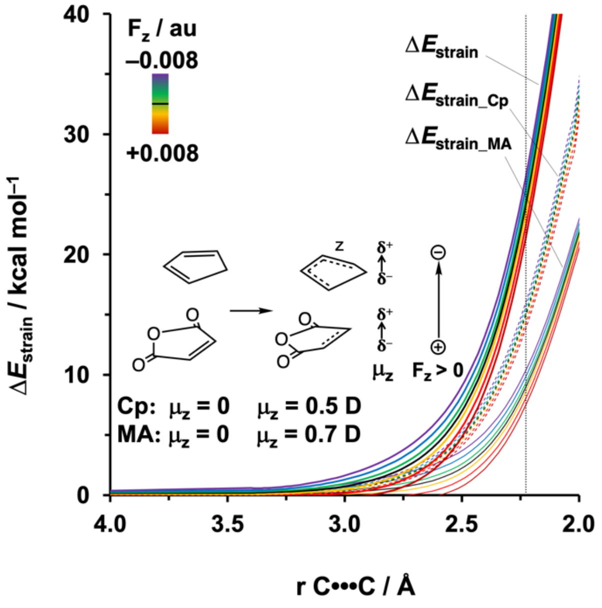

Figure S5. Decomposition of the ΔE_{strain} of the *endo* Diels-Alder reactions between **Cp** with **MA** under the F_z ranging from –0.008 to 0.008 au, projected onto the newly forming bond length. All results were computed at BP86/TZ2P.

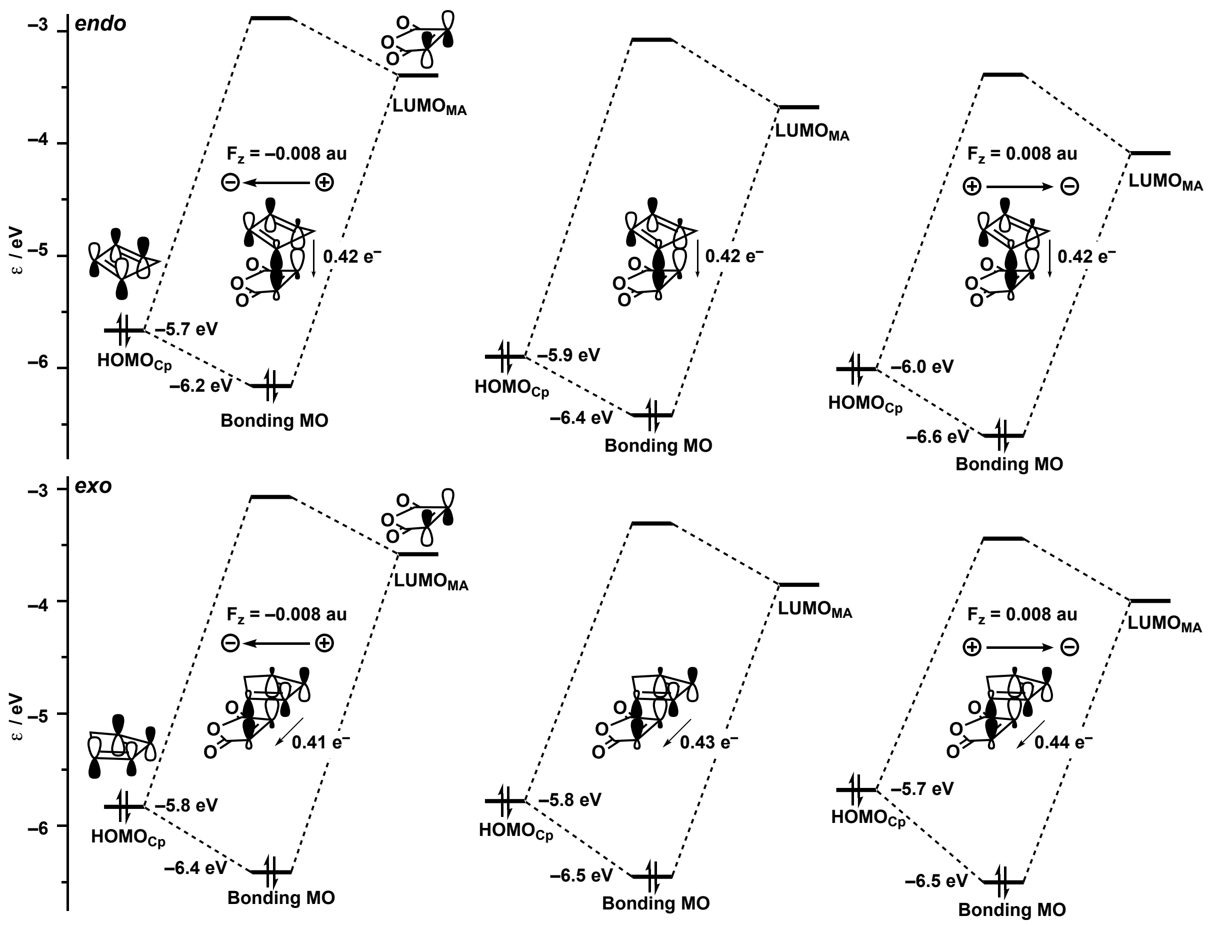

Figure S6. Diagrams of the NED interactions for the *endo* (top) and *exo* (bottom) Diels-Alder reactions between **Cp** and **MA** under a F_y at –0.008 au (left), 0 au (middle), and 0.008 au (right), computed at the transition states structures at BP86/TZ2P.

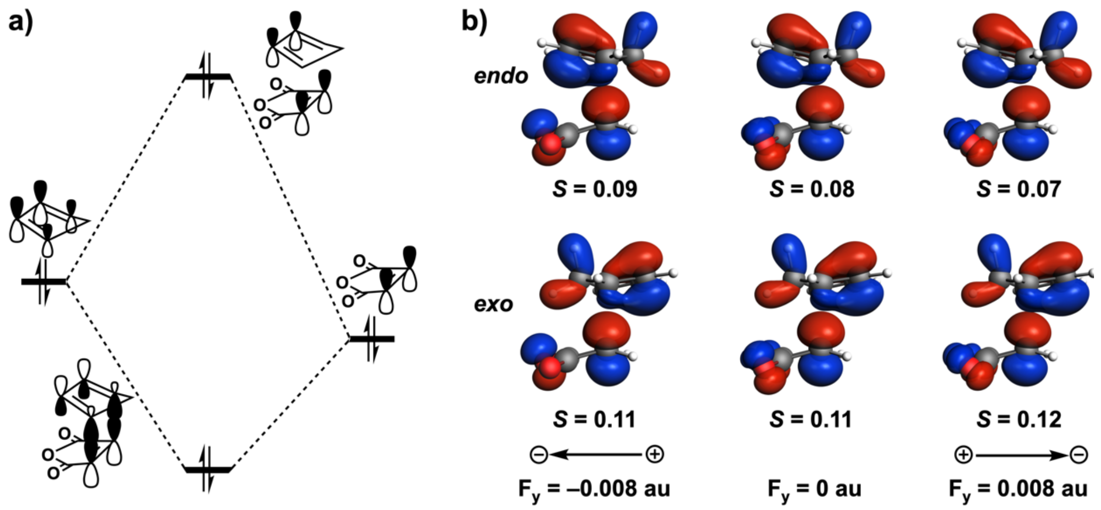

Figure S7. a) Schematic diagram for the HOMO–1$_{\mathbf{Cp}}$–HOMO–2$_{\mathbf{MA}}$ interaction of the Diels-Alder reaction between **Cp** and **MA** (*endo* as the example); b) the HOMO–1$_{\mathbf{Cp}}$|HOMO–2$_{\mathbf{MA}}$ overlaps and computed MOs of isolated reactants (isovalue = 0.06 Bohr$^{-3/2}$) for the *endo* and *exo* reactions under the F_y at –0.008 au, 0 au, and 0.008 au, where F_y is perpendicular to the reaction axis, *i.e.*, perpendicular to the plane of the newly forming C–C bonds, computed at the transition state structures at BP86/TZ2P.

The key two-center four-electron interaction have been quantified for the *endo* and *exo* Diels-Alder reactions between **Cp** and **MA** under a F_y of –0.008 au, 0 au, and 0.008 au. In all cases, the most profound interaction occurs between the HOMO–1$_{\mathbf{Cp}}$ and HOMO–2$_{\mathbf{MA}}$, which is predominantly located on the C=C double bonds of **Cp** and the C=C double bond of the five-membered ring of **MA**, respectively (Figure S7a). Since the shape of a MO can be modified by an external electric field,[1] the HOMO–1$_{\mathbf{Cp}}$, an orbital delocalized along the y-direction, is polarized by a positive F_y towards the C^{sp2}–C^{sp2} single bond for the *endo* pathway (Figure S7b). Consequently, the orbital overlap of the HOMO–1$_{\mathbf{Cp}}$|HOMO–2$_{\mathbf{MA}}$ in the reactive region, along the *endo* pathway, decreases from S = 0.09 to S = 0.07. On the other hand, along the *exo* pathway, a positive field polarized the HOMO–1$_{\mathbf{Cp}}$ towards the methylene bridge, increasing the overlap from S = 0.11 to S = 0.12, when F_y goes from –0.008 to 0.008 au. Note that the spatial distribution of the HOMO–2$_{\mathbf{MA}}$ remains nearly unchanged upon applying an electric field and for that reason has no effect on the observed change in orbital overlap. As a result, the ΔE_{Pauli} term for the *endo* pathway is getting less destabilizing while that for the *exo* pathway is becoming slightly more destabilizing going from a negative to positive F_y.

[1] a) H. Akagi, T. Otobe, R. Itakura, *Sci. Adv.* **2019**, *5*, eaaw1885; b) P. M. Kraus, O. I. Tolstikhin, D. Baykusheva, A. Rupenyan, J. Schneider, C. Z. Bisgaard, T. Morishita, F. Jensen, L. B. Madsen & H. J. Wörner, *Nat. Comm.* **2015**, *6*, 7039.

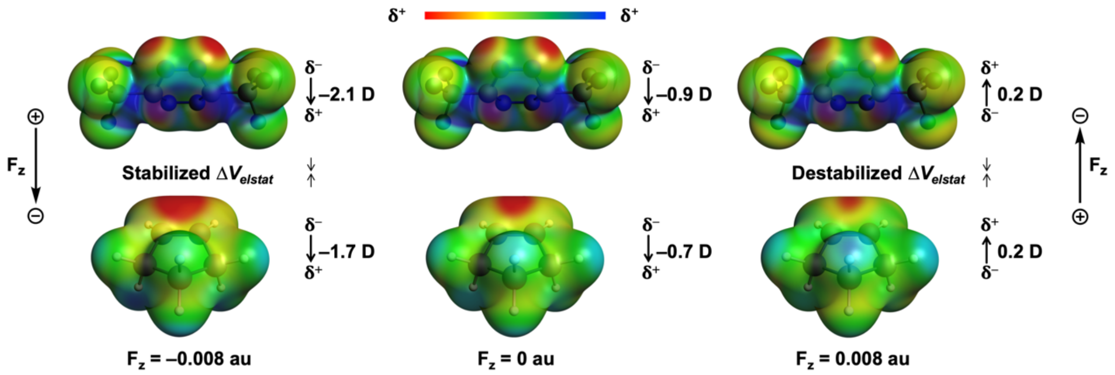

Figure S8. Molecular electrostatic potential maps (at 0.01 Bohr^{-3}) from −0.03 (red) to 0.1 (blue) Hartree e^{-1} and the dipole moments (μ_z, D) of isolated reactants for the Diels-Alder reactions between **Tz** and **Ce** under the F_z at –0.008, 0, and 0.008 au, where F_z is aligned along the reaction axis, i.e., along the axis of a newly forming C–C bond, computed at transition states at BP86/TZ2P.

Table S3. Cartesian coordinates (in Å), energies (***E***, ***G*** in kcal mol^{-1}), number of imaginary frequencies (N_{imag}), and the wavelength of the imaginary frequency (v, i cm^{-1}), of all stationary points, computed at BP86/TZ2P.

Cp_F=0
E = -1502.37
G = -1462.87
N_{imag} = 0

C	1.180907	0.001631	1.341206
C	-1.180857	0.001571	1.341234
C	0.732934	-1.275816	1.368461
C	-0.732818	-1.275853	1.368478
H	1.350690	-2.172058	1.387498
H	-1.350528	-2.172126	1.387530
C	0.000001	0.931850	1.321465
H	-0.000026	1.581402	0.428204
H	-0.000006	1.618391	2.186660
H	2.217056	0.330683	1.334233
H	-2.217023	0.330570	1.334285

MA_F=0
E = -1434.79
G = -1419.03
N_{imag} = 0

C	0.000000	-1.138419	-0.399175
C	0.000000	-0.668617	1.014977
C	0.000000	1.138419	-0.399175
C	0.000000	0.668617	1.014977
O	0.000000	0.000000	-1.224446
O	0.000000	-2.254441	-0.842186
O	0.000000	2.254441	-0.842186
H	0.000000	-1.361833	1.851527
H	0.000000	1.361833	1.851527

TS_*endo*_F=0
E = -2927.54
G = -2857.91
N_{imag} = 1, v = -376.051798

C	1.156811	-0.002070	1.094017
C	0.700231	-0.011815	-1.084564
C	-0.700335	-0.011864	-1.084486
C	-1.156761	-0.002117	1.094018
C	0.700857	-1.281562	1.452380
C	-0.700747	-1.281591	1.452390
H	1.331133	-2.157990	1.583794
H	-1.330986	-2.158045	1.583818
C	0.000008	0.946357	1.290653
H	-0.000009	1.848614	0.671253
H	-0.000003	1.267081	2.350013
H	2.200578	0.304041	1.113746
H	-2.200540	0.303953	1.113832
O	0.000008	-2.179789	-1.585701
O	2.251006	-1.799105	-1.695528
O	-2.251032	-1.799268	-1.695354
C	1.149418	-1.363602	-1.479258
C	-1.149457	-1.363683	-1.479169
H	1.347540	0.840995	-1.264554
H	-1.347713	0.840896	-1.264484

TS_*exo*_F=0
E = -2926.66
G = -2857.12
N_{imag} = 1, v = -375.453358

C	-1.157262	-0.002929	1.101327
C	-0.700813	-0.005929	-1.098538
C	0.700636	-0.005863	-1.098659
C	1.157434	-0.002807	1.101167
C	0.700838	1.302216	1.354288
C	-0.700767	1.302142	1.354393
H	1.331702	2.186488	1.418470
H	-1.331714	2.186348	1.418667
C	0.000154	-0.927058	1.368787
H	0.000171	-1.887061	0.844817
H	0.000240	-1.152926	2.452473
H	-1.348714	0.834793	-1.318812
H	2.198528	-0.316934	1.133832
H	-2.198318	-0.317165	1.134168
O	-0.000015	-2.190047	-1.566039
O	2.252702	-1.813942	-1.639435
O	-2.252789	-1.814163	-1.639074
C	1.148408	-1.370187	-1.454974
C	-1.148517	-1.370289	-1.454786
H	1.348418	0.834925	-1.319026

Cp_F=0_COSMO(DCM)
E = -1503.44
G = -1464.08
N_{imag} = 0

C	1.180649	0.002559	1.341174
C	-1.180599	0.002500	1.341219
C	0.733589	-1.277158	1.368477
C	-0.733472	-1.277193	1.368528
H	1.350488	-2.174491	1.387634
H	-1.350325	-2.174561	1.387505
C	0.000001	0.932083	1.321460
H	-0.000039	1.584882	0.430530

H	0.000007	1.621700	2.184254
H	2.216785	0.333247	1.334207
H	-2.216752	0.333130	1.334115

MA_F=0_COSMO(DCM)

E = -1443.15

G = -1427.55

N_{imag} = 0

C	0.000000	-1.133275	-0.391125
C	0.000000	-0.668973	1.017180
C	0.000000	1.133275	-0.391125
C	0.000000	0.668973	1.017180
O	0.000000	0.000000	-1.221338
O	0.000000	-2.247415	-0.851655
O	0.000000	2.247415	-0.851655
H	0.000000	-1.358876	1.855073
H	0.000000	1.358876	1.855073

TS_*endo*_F=0_COSMO(DCM)

E = -2937.81

G = -2868.53

N_{imag} = 1, v = -354.515043

C	1.156268	-0.002014	1.095310
C	0.702524	-0.011813	-1.082316
C	-0.702606	-0.011824	-1.082319
C	-1.156199	-0.001959	1.095578
C	0.700193	-1.283766	1.455285
C	-0.700128	-1.283724	1.455444
H	1.331430	-2.156793	1.604466
H	-1.331384	-2.156720	1.604734
C	0.000071	0.945960	1.282877
H	0.000017	1.847123	0.663592
H	0.000201	1.270213	2.340915
H	2.198925	0.306898	1.112356
H	-2.198840	0.306998	1.112701
O	-0.000020	-2.176712	-1.573796
O	2.243731	-1.807623	-1.707716
O	-2.243775	-1.807656	-1.707722
C	1.145274	-1.352885	-1.474944
C	-1.145329	-1.352887	-1.474952
H	1.345364	0.844254	-1.258569
H	-1.345475	0.844242	-1.258458

TS_*exo*_F=0_COSMO(DCM)

E = -2936.13

G = -2867.17

N_{imag} = 1, v = -349.363495

C	-1.206309	-0.107370	0.743959
C	-0.807602	0.048468	-1.469967
C	0.597519	0.039538	-1.505375
C	1.106693	-0.121461	0.683231
C	0.666441	1.171863	1.019314
C	-0.733436	1.180386	1.055906
H	1.306576	2.045441	1.120808
H	-1.356716	2.061670	1.190372
C	-0.050188	-1.052262	0.918664
H	-0.071129	-1.978789	0.338534
H	-0.023998	-1.349983	1.983926
H	-1.450416	0.909617	-1.608645
H	2.146623	-0.439882	0.673861
H	-2.249141	-0.413090	0.788069
O	-0.134338	-2.096008	-2.109362
O	2.111243	-1.738860	-2.234962
O	-2.378689	-1.710218	-2.121942
C	1.018543	-1.286581	-1.971887
C	-1.268600	-1.271785	-1.914053
H	1.243203	0.892418	-1.677847

Cp_F$_x$=-/+0.008

E = -1503.67

G = -1464.19

N_{imag} = 0

C	0.002110	0.001619	0.000000
C	1.467410	-0.000509	0.000000
C	1.912315	1.277477	0.000000
C	-0.450143	1.279663	0.000000
C	0.733158	2.209004	0.000000
H	2.947567	1.608269	0.000000
H	2.088802	-0.893558	0.000000
H	-0.612635	-0.898325	0.000000
H	-1.488423	1.606435	0.000000
H	0.742715	2.877048	-0.879508
H	0.742715	2.877048	0.879508

MA_F$_x$=-/+0.008

E = -1436.74

G = -1421.51

N_{imag} = 0

C	0.002407	0.007117	0.000000
C	1.339795	-0.007254	0.000000
C	1.821249	1.403419	0.000000
C	-0.459090	1.423209	0.000000
O	0.654549	2.240410	0.000000
O	2.922809	1.857359	0.000000
O	-1.590770	1.853881	0.000000
H	2.028458	-0.848217	0.000000
H	-0.695438	-0.825496	0.000000

TS_*endo*_F$_x$=-0.008

E = -2930.53

G = -2860.93
N_{imag} = 1, v = -375.803041

C	-0.003420	-0.001680	-0.000669
C	1.397953	-0.000461	0.000007
C	-0.422223	2.219276	0.016639
H	-0.648989	-0.186812	0.852042
C	1.882897	2.125678	0.193771
C	1.540193	2.561016	-1.099598
C	0.143410	2.611805	-1.206225
H	2.247726	2.716083	-1.910820
H	-0.404963	2.797203	-2.127153
C	0.662293	2.329678	1.059026
H	0.572715	1.673399	1.930297
H	0.688682	3.373531	1.426073
H	2.899041	2.111583	0.582454
H	-1.487588	2.247396	0.238461
O	0.647317	-0.408707	-2.188534
O	2.914851	-0.535845	-1.848591
O	-1.590648	-0.531889	-1.774264
C	1.835316	-0.336368	-1.377704
C	-0.466463	-0.336948	-1.357209
H	2.046131	-0.253737	0.834264

TS_*exo*_F$_x$=-0.008
E = -2929.71
G = -2860.16
N_{imag} = 1, v = -375.762615

C	-0.003371	0.000785	-0.000749
C	1.398625	-0.003720	-0.000828
C	-0.404505	2.259795	0.013546
H	2.046103	-0.291321	0.820615
C	1.904056	2.132728	0.130509
C	1.391151	2.371042	1.420452
C	-0.006545	2.444226	1.346995
H	1.979307	2.381932	2.335491
H	-0.679067	2.507490	2.201024
C	0.809355	2.493118	-0.841065
H	0.832518	2.002090	-1.818073
H	0.888461	3.581439	-1.027675
H	-0.650242	-0.204784	0.844137
H	2.961955	2.146938	-0.124155
H	-1.425570	2.332880	-0.356224
O	0.643426	-0.400354	-2.198940
O	2.912567	-0.495722	-1.863201
O	-1.596226	-0.474934	-1.784272
C	1.831291	-0.319371	-1.386674
C	-0.468463	-0.310025	-1.363284

TS_*endo*_F$_x$=+0.008
E = -2930.53

G = -2860.93
N_{imag} = 1, v = -375.803023

C	-0.003535	-0.001458	0.000607
C	1.397835	-0.000485	-0.000088
C	-0.421975	2.219536	-0.016571
H	-0.649150	-0.186391	-0.852114
C	1.883146	2.125690	-0.193587
C	1.540427	2.560935	1.099800
C	0.143640	2.611897	1.206351
H	2.247933	2.715844	1.911076
H	-0.404761	2.797302	2.127261
C	0.662614	2.329883	-1.058892
H	0.573007	1.673672	-1.930211
H	0.689129	3.373758	-1.425868
H	2.899309	2.111438	-0.582215
H	-1.487320	2.247836	-0.238462
O	0.647153	-0.408774	2.188427
O	2.914631	-0.536680	1.848355
O	-1.590846	-0.531441	1.774198
C	1.835158	-0.336687	1.377548
C	-0.466621	-0.336720	1.357139
H	2.045959	-0.253715	-0.834399

TS_*exo*_F$_x$=+0.008
E = -2929.71
G = -2860.16
N_{imag} = 1, v = -375.717557

C	-0.003319	0.000643	0.000783
C	1.398683	-0.003542	0.000889
C	-0.404589	2.260043	-0.013686
H	2.046169	-0.291261	-0.820511
C	1.903963	2.132582	-0.130477
C	1.391165	2.370920	-1.420477
C	-0.006529	2.444257	-1.347116
H	1.979383	2.381693	-2.335477
H	-0.678987	2.507544	-2.201193
C	0.809235	2.493213	0.840998
H	0.832228	2.002221	1.818027
H	0.888517	3.581530	1.027557
H	-0.650172	-0.204894	-0.844121
H	2.961852	2.146845	0.124235
H	-1.425672	2.333136	0.356027
O	0.643489	-0.400099	2.199024
O	2.912549	-0.496373	1.863110
O	-1.596181	-0.474735	1.784384
C	1.831314	-0.319384	1.386730
C	-0.468428	-0.309906	1.363337

Cp_F$_x$=-/+0.008_COSMO(DCM)
E = -1505.23

G = -1466.02
Nimag = 0

C	0.002353	0.000334	0.000000
C	1.468277	-0.002204	0.000000
C	1.911174	1.278163	0.000000
C	-0.451165	1.281126	0.000000
C	0.733397	2.209311	0.000000
H	2.945981	1.611423	0.000000
H	2.090207	-0.895120	0.000000
H	-0.610258	-0.902061	0.000000
H	-1.490428	1.608167	0.000000
H	0.747475	2.880384	-0.877208
H	0.747475	2.880384	0.877208

MA_F$_x$=-/+0.008_COSMO(DCM)
E = -1446.27
G = -1431.32
Nimag = 0

C	0.002645	0.009292	0.000000
C	1.340893	-0.012307	0.000000
C	1.827097	1.389454	0.000000
C	-0.448033	1.419938	0.000000
O	0.645072	2.239887	0.000000
O	2.917055	1.866022	0.000000
O	-1.589019	1.861245	0.000000
H	2.023516	-0.856985	0.000000
H	-0.693740	-0.823304	0.000000

TS_*endo*_F$_x$=-0.008_COSMO(DCM)
E = -2942.83
G = -2874.17
Nimag = 1, *v* = -338.828274

C	0.002797	-0.045530	0.007100
C	1.408293	0.029885	-0.003743
C	-0.426656	2.308189	-0.021902
H	-0.630912	-0.194512	0.873966
C	1.861840	2.058824	0.227149
C	1.581819	2.530085	-1.076844
C	0.192724	2.650514	-1.228384
H	2.326664	2.676453	-1.855432
H	-0.312191	2.878499	-2.164865
C	0.624175	2.327193	1.052812
H	0.463234	1.672426	1.913598
H	0.715438	3.361646	1.435106
H	2.862636	2.034818	0.652593
H	-1.498330	2.349576	0.165096
O	0.627338	-0.398695	-2.184853
O	2.899406	-0.500297	-1.887829
O	-1.594740	-0.593689	-1.752312
C	1.833059	-0.306227	-1.385506

C	-0.461780	-0.363250	-1.325343
H	2.062738	-0.252160	0.817152

TS_*exo*_F$_x$=-0.008_COSMO(DCM)
E = -2941.44
G = -2873.26
Nimag = 1, *v* = -326.819728

C	-0.003838	-0.064316	0.001279
C	1.401060	0.023281	-0.001231
C	-0.382496	2.441313	-0.018093
H	2.054286	-0.308202	0.800403
C	1.893561	2.026907	0.157843
C	1.361260	2.296280	1.441532
C	-0.021820	2.506931	1.328191
H	1.921605	2.239803	2.371706
H	-0.711931	2.616552	2.163039
C	0.864422	2.518170	-0.835605
H	0.844807	2.037654	-1.817841
H	1.094049	3.585403	-1.018828
H	-0.646944	-0.195767	0.862677
H	2.961412	2.017280	-0.051891
H	-1.385279	2.574914	-0.419507
O	0.631047	-0.403148	-2.195880
O	2.900216	-0.431496	-1.898958
O	-1.597678	-0.571457	-1.771096
C	1.824682	-0.284897	-1.398864
C	-0.465334	-0.357637	-1.331360

TS_*endo*_F$_x$=+0.008_COSMO(DCM)
E = -2942.83
G = -2874.17
Nimag = 1, *v* = -363.655728

C	0.002797	-0.045530	0.007100
C	1.408293	0.029885	-0.003743
C	-0.426656	2.308189	-0.021902
H	-0.630912	-0.194512	0.873966
C	1.861840	2.058824	0.227149
C	1.581819	2.530085	-1.076844
C	0.192724	2.650514	-1.228384
H	2.326664	2.676453	-1.855432
H	-0.312191	2.878499	-2.164865
C	0.624175	2.327193	1.052812
H	0.463234	1.672426	1.913598
H	0.715438	3.361646	1.435106
H	2.862636	2.034818	0.652593
H	-1.498330	2.349576	0.165096
O	0.627338	-0.398695	-2.184853
O	2.899406	-0.500297	-1.887829
O	-1.594740	-0.593689	-1.752312
C	1.833059	-0.306227	-1.385506

C	-0.461780	-0.363250	-1.325343
H	2.062738	-0.252160	0.817152

TS_*exo*_F_x=+0.008_COSMO(DCM)

E = -2942.18
G = -2873.39
N_{imag} = 1, ν = -323.059751

C	-0.000136	-0.000980	0.000195
C	1.408083	0.000347	0.000280
C	-0.195516	2.562890	-0.000297
H	2.036327	-0.383553	-0.798096
C	2.037321	1.952601	-0.176123
C	1.523736	2.250392	-1.461568
C	0.162568	2.575718	-1.347400
H	2.075163	2.140832	-2.392360
H	-0.518338	2.735003	-2.181682
C	1.053956	2.536270	0.814227
H	0.993434	2.066823	1.800259
H	1.375424	3.580910	0.990273
H	-0.652611	-0.082367	-0.860365
H	3.102663	1.867426	0.029664
H	-1.182861	2.780626	0.402340
O	0.616742	-0.363871	2.198222
O	2.877184	-0.545164	1.898128
O	-1.619877	-0.399471	1.776599
C	1.811876	-0.328192	1.400645
C	-0.477497	-0.254896	1.332257

Cp_F_y=-0.008(*endo*)/+0.008(*exo*)

E = -1502.68
G = -1462.76
N_{imag} = 0

C	-0.732991	0.000000	0.001069
C	0.732991	0.000000	0.001069
C	1.181886	0.000000	1.277620
C	-1.181886	0.000000	1.277620
C	0.000000	0.000000	2.211551
H	2.220102	0.000000	1.601386
H	1.345615	0.000000	-0.898179
H	-1.345615	0.000000	-0.898179
H	-2.220102	0.000000	1.601386
H	0.000000	0.884072	2.875279
H	0.000000	-0.884072	2.875279

Cp_F_y=+0.008(*endo*)/–0.008(*exo*)

E = -1504.82
G = -1464.96
N_{imag} = 0

C	-0.733061	0.000000	-0.000081
C	0.733061	0.000000	-0.000081
C	1.180022	0.000000	1.279481
C	-1.180022	0.000000	1.279481
C	0.000000	0.000000	2.206309
H	2.213925	0.000000	1.614981
H	1.355674	0.000000	-0.894856
H	-1.355674	0.000000	-0.894856
H	-2.213925	0.000000	1.614981
H	0.000000	0.874319	2.881100
H	0.000000	-0.874319	2.881100

MA_F_y=-0.008

E = -1427.72
G = -1412.03
N_{imag} = 0

C	0.000000	0.667808	0.002740
C	0.000000	-0.667808	0.002740
C	0.000000	-1.137070	1.421162
C	0.000000	1.137070	1.421162
O	0.000000	0.000000	2.240493
O	0.000000	-2.258077	1.847070
O	0.000000	2.258077	1.847070
H	0.000000	-1.367393	-0.828707
H	0.000000	1.367393	-0.828707

MA_F_y=+0.008

E = -1444.10
G = -1428.29
N_{imag} = 0

C	0.000000	0.669619	-0.004713
C	0.000000	-0.669619	-0.004713
C	0.000000	-1.140026	1.406012
C	0.000000	1.140026	1.406012
O	0.000000	0.000000	2.237302
O	0.000000	-2.250726	1.866787
O	0.000000	2.250726	1.866787
H	0.000000	-1.356469	-0.847190
H	0.000000	1.356469	-0.847190

TS_*endo*_F_y=-0.008

E = -2920.37
G = -2850.9
N_{imag} = 1, ν = -380.125935

C	-0.002043	0.000910	-0.000035
C	1.395827	-0.002232	-0.002422
C	-0.455655	2.183267	-0.000876
H	-0.656950	-0.175248	-0.848183
C	1.859203	2.178074	-0.004546
C	1.406168	2.548293	1.271051
C	0.003074	2.551429	1.273281
H	2.034838	2.689319	2.147240

H	-0.622199	2.695274	2.151442
C	0.700690	2.371970	-0.955020
H	0.697843	1.733591	-1.846323
H	0.702514	3.427422	-1.288624
H	2.903865	2.195179	-0.308789
H	-1.501184	2.205010	-0.301815
O	0.699513	-0.516873	2.163826
O	2.951106	-0.639321	1.763061
O	-1.554009	-0.629313	1.770844
C	1.845742	-0.411340	1.349879
C	-0.449138	-0.406203	1.353805
H	2.047031	-0.181289	-0.852815

TS_*exo*_F_y=-0.008

E = -2919.63

G = -2850.22

N$_{imag}$ = 1, *v* = -388.411487

C	-0.000482	-0.002749	-0.001355
C	1.397888	-0.002877	-0.001424
C	-0.458534	2.194062	-0.000341
H	2.050482	-0.224384	-0.838360
C	1.856470	2.193660	-0.000477
C	1.400148	2.435481	-1.308395
C	-0.002290	2.435699	-1.308313
H	2.033629	2.482634	-2.193218
H	-0.635860	2.483050	-2.193062
C	0.699064	2.465539	0.919928
H	0.699029	1.954836	1.887052
H	0.699268	3.550869	1.141350
H	-0.653218	-0.224040	-0.838236
H	2.895934	2.228324	0.318815
H	-1.497950	2.228994	0.319075
O	0.698763	-0.481974	2.181199
O	2.952713	-0.568810	1.788499
O	-1.555256	-0.568291	1.788733
C	1.844993	-0.372351	1.366573
C	-0.447542	-0.372092	1.366676

TS_*endo*_F_y=+0.008

E = -2939.01

G = -2869.31

N$_{imag}$ = 1, *v* = -375.038791

C	0.002093	-0.002438	-0.000150
C	1.406303	0.000797	0.001918
C	-0.456936	2.169074	0.001157
H	-0.637065	-0.179745	-0.860094
C	1.855244	2.174496	0.004779
C	1.396324	2.508707	1.292369
C	-0.003623	2.505420	1.290179
H	2.027953	2.619790	2.171218

H	-0.638530	2.613561	2.167026
C	0.700132	2.384767	-0.939628
H	0.702944	1.795106	-1.860457
H	0.698077	3.451605	-1.236696
H	2.898106	2.200628	-0.303597
H	-1.498934	2.190403	-0.310493
O	0.702267	-0.511737	2.166590
O	2.952341	-0.604832	1.805019
O	-1.546329	-0.615166	1.798530
C	1.854853	-0.395173	1.349293
C	-0.448560	-0.400473	1.345925
H	2.048779	-0.173479	-0.856166

TS_*exo*_F_y=+0.008

E = -2938.44

G = -2872.59

N$_{imag}$ = 1, *v* = -357.677967

C	0.000017	-0.000334	-0.000008
C	1.405520	0.000417	0.000052
C	-0.455527	2.200047	-0.000009
H	2.047840	-0.215863	-0.846463
C	1.858702	2.201310	0.000097
C	1.402097	2.472731	-1.301559
C	0.000903	2.471970	-1.301623
H	2.030289	2.558305	-2.185607
H	-0.627301	2.556868	-2.185728
C	0.701403	2.461184	0.929556
H	0.701656	1.919072	1.880135
H	0.700802	3.541948	1.166795
H	-0.641997	-0.217313	-0.846576
H	2.901304	2.231255	0.309887
H	-1.498190	2.228889	0.309684
O	0.702925	-0.468871	2.183567
O	2.954344	-0.526741	1.824248
O	-1.548392	-0.529226	1.824037
C	1.853876	-0.351168	1.359167
C	-0.448072	-0.352428	1.359069

Cp_F_y=-0.008(*endo*)/+0.008(*exo*) _COSMO(DCM)

E = -1503.77

G = -1464.09

N$_{imag}$ = 0

C	-0.733432	0.000000	-0.000111
C	0.733432	0.000000	-0.000111
C	1.182101	0.000000	1.278036
C	-1.182101	0.000000	1.278036
C	0.000000	0.000000	2.213246
H	2.221224	0.000000	1.601094
H	1.342982	0.000000	-0.901689

H	-1.342982	0.000000	-0.901689
H	-2.221224	0.000000	1.601094
H	0.000000	0.883570	2.878098
H	0.000000	-0.883570	2.878098

Cp_F$_y$=+0.008(*endo*)/-0.008(*exo*) _COSMO(DCM)

E = -1506.99

G = -1467.44

N$_{imag}$ = 0

C	-0.734171	0.000000	-0.001922
C	0.734171	0.000000	-0.001922
C	1.179328	0.000000	1.281205
C	-1.179328	0.000000	1.281205
C	0.000000	0.000000	2.204904
H	2.212127	0.000000	1.621389
H	1.358167	0.000000	-0.896921
H	-1.358167	0.000000	-0.896921
H	-2.212127	0.000000	1.621389
H	0.000000	0.869510	2.886521
H	0.000000	-0.869510	2.886521

MA_F$_y$=-0.008_COSMO(DCM)

E = -1433.89

G = -1418.27

N$_{imag}$ = 0

C	0.000000	0.667778	0.002122
C	0.000000	-0.667778	0.002122
C	0.000000	-1.131120	1.416247
C	0.000000	1.131120	1.416247
O	0.000000	0.000000	2.238157
O	0.000000	-2.252480	1.852416
O	0.000000	2.252480	1.852416
H	0.000000	-1.365978	-0.829129
H	0.000000	1.365978	-0.829129

MA_F$_y$=+0.008_COSMO(DCM)

E = -1455.48

G = -1439.87

N$_{imag}$ = 0

C	0.000000	0.670505	-0.008750
C	0.000000	-0.670505	-0.008750
C	0.000000	-1.135808	1.394821
C	0.000000	1.135808	1.394821
O	0.000000	0.000000	2.233743
O	0.000000	-2.242293	1.880443
O	0.000000	2.242293	1.880443
H	0.000000	-1.352166	-0.854352
H	0.000000	1.352166	-0.854352

TS_*endo*_F$_y$=-0.008_COSMO(DCM)

E = -2927.75

G = -2858.59

N$_{imag}$ = 1, *ν* = -359.611540

C	-0.000347	-0.000801	-0.001705
C	1.400497	-0.000789	-0.001685
C	-0.457315	2.182220	-0.000780
H	-0.651414	-0.175896	-0.852033
C	1.857337	2.182270	-0.000768
C	1.401315	2.555908	1.274125
C	-0.001327	2.555893	1.274120
H	2.027381	2.716149	2.148753
H	-0.627415	2.716106	2.148738
C	0.700012	2.363701	-0.954331
H	0.700027	1.719593	-1.841131
H	0.699989	3.416486	-1.294866
H	2.902024	2.199896	-0.304455
H	-1.501999	2.199787	-0.304483
O	0.700049	-0.515085	2.159202
O	2.945730	-0.657928	1.765992
O	-1.545627	-0.657895	1.765943
C	1.841987	-0.412571	1.341739
C	-0.441872	-0.412554	1.341710
H	2.051599	-0.175844	-0.851994

TS_*exo*_F$_y$=-0.008_COSMO(DCM)

E = -2926.49

G = -2857.51

N$_{imag}$ = 1, *ν* = -373.754113

C	-0.000647	-0.004250	-0.001955
C	1.400100	-0.004148	-0.001965
C	-0.457983	2.196327	-0.000279
H	2.051383	-0.227646	-0.838726
C	1.856245	2.197767	-0.000092
C	1.400096	2.413709	-1.314522
C	-0.001828	2.412892	-1.314644
H	2.035726	2.444691	-2.198796
H	-0.637338	2.443158	-2.199029
C	0.698912	2.474021	0.914771
H	0.699171	1.980271	1.889976
H	0.698201	3.560598	1.129917
H	-0.651845	-0.228423	-0.838607
H	2.895472	2.240840	0.317856
H	-1.497308	2.238585	0.317466
O	0.699824	-0.485244	2.176129
O	2.946844	-0.598524	1.785754
O	-1.547193	-0.598671	1.785958
C	1.840128	-0.380023	1.357369
C	-0.440505	-0.380112	1.357539

TS_*endo*_F_y=+0.008_COSMO(DCM)
E = -2954.13
G = -2885.01
Nimag = 1, *v* = -351.847620

C	0.001077	-0.002079	0.001597
C	1.412508	-0.001666	0.001692
C	-0.450117	2.160772	0.001527
H	-0.631674	-0.174671	-0.863743
C	1.859820	2.164919	0.002834
C	1.402368	2.497848	1.296426
C	0.004872	2.495660	1.295635
H	2.037904	2.624863	2.171040
H	-0.632093	2.620770	2.169482
C	0.705079	2.369708	-0.937945
H	0.706682	1.787569	-1.862299
H	0.703294	3.437121	-1.234257
H	2.900572	2.187229	-0.311479
H	-1.490564	2.180781	-0.313973
O	0.706889	-0.500299	2.164580
O	2.948548	-0.620822	1.819176
O	-1.534477	-0.623094	1.818962
C	1.856133	-0.394077	1.335740
C	-0.442162	-0.395239	1.335882
H	2.045786	-0.172108	-0.863664

TS_*exo*_F_y=+0.008_COSMO(DCM)
E = -2954.23
G = -2884.17
Nimag = 1, *v* = -303.221777

C	-0.035422	0.013550	0.049214
C	1.375808	0.012715	-0.007853
C	-0.680252	1.961216	-0.141046
C	1.536927	2.639401	-0.320331
C	1.002756	2.547636	-1.599497
C	-0.353952	2.192807	-1.499492
H	1.549421	2.677665	-2.530035
H	-1.021706	2.026606	-2.341786
C	0.416456	2.621966	0.670237
H	0.645791	2.184795	1.647868
H	0.100015	3.666243	0.850252
H	2.567965	2.888285	-0.081301
H	-1.709189	1.887815	0.207543
O	0.761139	-0.216270	2.235461
O	3.012671	-0.232562	1.803483
O	-1.473864	-0.438116	1.999637
C	1.900723	-0.136296	1.309965
C	-0.394386	-0.241371	1.469095
H	1.993799	-0.099777	-0.891030
H	-0.679108	-0.399433	-0.721853

Cp_F_z=-/+0.008
E = -1503.28
G = -1463.78
Nimag = 0

C	0.000381	0.000648	0.732872
C	0.000381	0.000648	-0.732872
C	-0.010190	1.278579	-1.181058
C	-0.010190	1.278579	1.181058
C	0.001729	2.208702	0.000000
H	0.027960	1.607345	-2.216688
H	0.046097	-0.894909	-1.350305
H	0.046097	-0.894909	1.350305
H	0.027960	1.607345	2.216688
H	-0.846116	2.916860	0.000000
H	0.910987	2.836362	0.000000

MA_F_z=-/+0.008
E = -1435.47
G = -1420.2
Nimag = 0

C	0.000435	-0.000768	0.003731
C	1.337745	-0.000770	0.003731
C	1.807141	1.413587	0.014349
C	-0.468955	1.413596	0.014349
O	0.669086	2.238305	0.044197
O	2.922100	1.857011	-0.035199
O	-1.583921	1.857009	-0.035202
H	2.031092	-0.837191	-0.007340
H	-0.692914	-0.837186	-0.007339

TS_*endo*_F_z=-0.008
E = -2922.54
G = -2852.84
Nimag = 1, *v* = -400.998530

C	-0.000786	0.004376	0.003410
C	1.396649	0.006469	0.004570
C	-0.465431	2.176491	0.001953
H	-0.646544	-0.195064	-0.846923
C	1.848718	2.183260	-0.004601
C	1.395938	2.534587	1.278253
C	-0.007565	2.529775	1.282390
H	2.025121	2.620203	2.161661
H	-0.632126	2.611363	2.169474
C	0.688398	2.391120	-0.948659
H	0.687688	1.779849	-1.855759
H	0.684545	3.456132	-1.256335
H	2.892051	2.193698	-0.312955
H	-1.510558	2.180200	-0.300400
O	0.697073	-0.451228	2.183707
O	2.954800	-0.473992	1.824465

O	-1.558982	-0.503796	1.815513
C	1.848488	-0.339994	1.375683
C	-0.453246	-0.350643	1.371892
H	2.044923	-0.191292	-0.844212

TS_*exo*_F$_z$=-0.008
E = -2922.29
G = -2852.57
N$_{imag}$ = 1, *v* = -399.530862

C	-0.004470	0.012155	0.005286
C	1.393934	-0.002843	0.001759
C	-0.435288	2.218561	-0.000070
H	2.036250	-0.254072	-0.835193
C	1.879651	2.178306	-0.010323
C	1.422616	2.459285	-1.309454
C	0.018839	2.481971	-1.303311
H	2.047999	2.492361	-2.199547
H	-0.612950	2.534590	-2.187959
C	0.730443	2.465740	0.922442
H	0.725023	1.931438	1.876633
H	0.751027	3.547849	1.159118
H	-0.657261	-0.217874	-0.829542
H	2.921869	2.182234	0.302656
H	-1.474187	2.244829	0.322384
O	0.694672	-0.409785	2.200238
O	2.950942	-0.426316	1.836980
O	-1.563083	-0.376352	1.846155
C	1.843866	-0.311630	1.383962
C	-0.455538	-0.285322	1.388293

TS_*endo*_F$_z$=+0.008
E = -2938.47
G = -2872.96
N$_{imag}$ = 1, *v* = -340.046850

C	-0.000834	0.002582	-0.009010
C	1.405935	-0.008731	-0.011050
C	-0.455927	2.154910	0.006214
H	-0.644994	-0.152532	-0.869084
C	1.856117	2.193900	0.007333
C	1.394978	2.536746	1.289498
C	-0.004189	2.518772	1.288706
H	2.025416	2.712988	2.157607
H	-0.639938	2.680122	2.155876
C	0.698604	2.371676	-0.940354
H	0.710147	1.759244	-1.848031
H	0.680493	3.431743	-1.256841
H	2.899318	2.233891	-0.298647
H	-1.499155	2.187153	-0.301276
O	0.701816	-0.625310	2.125138
O	2.945434	-0.786138	1.721569

O	-1.539046	-0.787143	1.720797
C	1.851618	-0.488419	1.304597
C	-0.443639	-0.486993	1.309469
H	2.051349	-0.144949	-0.872811

TS_*exo*_F$_z$=+0.008
E = -2936.94
G = -2867.85
N$_{imag}$ = 1, *v* = -344.606970

C	-0.002419	-0.027569	-0.010614
C	1.404072	0.012608	-0.009590
C	-0.562092	2.093499	0.004490
H	2.061050	-0.126013	-0.859868
C	1.742550	2.303402	0.002099
C	1.269510	2.496450	-1.305940
C	-0.125109	2.384944	-1.304359
H	1.892831	2.634696	-2.186975
H	-0.763495	2.424455	-2.184745
C	0.570647	2.461642	0.924972
H	0.621689	1.947152	1.888949
H	0.470587	3.542735	1.144046
H	-0.634249	-0.274192	-0.856600
H	2.779133	2.410453	0.313267
H	-1.605003	2.081858	0.314852
O	0.716691	-0.566264	2.150830
O	2.968449	-0.625396	1.755471
O	-1.514865	-0.808025	1.745608
C	1.865006	-0.394786	1.322737
C	-0.426095	-0.488750	1.329852

Cp_F$_z$=-/+0.008_COSMO(DCM)
E = -1504.73
G = -1465.5
N$_{imag}$ = 0

C	0.000534	-0.000716	0.733508
C	0.000534	-0.000716	-0.733508
C	-0.013371	1.279628	-1.180910
C	-0.013371	1.279628	1.180910
C	0.001701	2.208738	0.000000
H	0.036468	1.609771	-2.216155
H	0.061498	-0.896682	-1.349793
H	0.061498	-0.896682	1.349793
H	0.036468	1.609771	2.216155
H	-0.830503	2.935085	0.000000
H	0.920012	2.823907	0.000000

MA_F$_z$=-/+0.008_COSMO(DCM)
E = -1444.20
G = -1428.99
N$_{imag}$ = 0

C	0.000000	0.000000	0.000000
C	1.338045	0.000000	0.000000
C	1.801598	1.408690	0.000000
C	-0.463560	1.408687	0.000174
O	0.669020	2.238319	0.031255
O	2.913817	1.869253	-0.071718
O	--1.575799	1.869249	-0.071253
H	2.028167	-0.837735	-0.009917
H	-0.690120	-0.837737	-0.009855

TS_*endo*_F_z=-0.008_COSMO(DCM)
E = -2931.43
G = -2861.9
N_{imag} = 1, v = -395.204049

C	-0.002553	0.009877	0.006010
C	1.396402	0.002128	0.004457
C	-0.456218	2.178164	-0.001783
H	-0.645946	-0.193413	-0.844452
C	1.857845	2.183325	-0.005561
C	1.404597	2.540029	1.277216
C	0.000237	2.538517	1.279590
H	2.032019	2.629570	2.162157
H	-0.624464	2.626590	2.166609
C	0.699188	2.384099	-0.951314
H	0.699200	1.769383	-1.854841
H	0.695938	3.447592	-1.265658
H	2.900382	2.183345	-0.316217
H	-1.499705	2.179405	-0.309405
O	0.697613	-0.420216	2.187336
O	2.949065	-0.456656	1.839171
O	-1.553290	-0.469251	1.837134
C	1.844435	-0.326543	1.371292
C	-0.449154	-0.326864	1.372166
H	2.038193	-0.200261	-0.847265

TS_*exo*_F_z=-0.008_COSMO(DCM)
E = -2930.35
G = -2860.93
N_{imag} = 1, v = -393.648754

C	-0.002984	0.008928	0.005799
C	1.397173	0.002926	0.004081
C	-0.443573	2.219474	0.000687
H	2.038489	-0.252103	-0.831917
C	1.871572	2.188026	-0.009230
C	1.413571	2.453574	-1.312241
C	0.009279	2.470525	-1.306057
H	2.038354	2.470198	-2.203090
H	-0.622600	2.502750	-2.191498
C	0.721126	2.476658	0.921652
H	0.717698	1.949733	1.879510

H	0.737517	3.558703	1.158205
H	-0.650270	-0.228747	-0.830563
H	2.914471	2.192318	0.301023
H	-1.483325	2.243823	0.320347
O	0.696886	-0.386758	2.202114
O	2.947516	-0.390240	1.855677
O	-1.554921	-0.366919	1.858959
C	1.841503	-0.289298	1.383289
C	-0.449058	-0.276735	1.383651

TS_*endo*_F_z=+0.008_COSMO(DCM)
E = -2954.26
G = -2886.32
N_{imag} = 1, v = -259.662484

C	0.038771	0.003863	0.043894
C	1.438665	-0.157889	0.039692
C	-0.498169	1.993883	0.010854
H	-0.596446	-0.168522	-0.821163
C	1.709489	2.748511	-0.090396
C	1.264818	2.871067	1.214702
C	-0.092868	2.492247	1.268641
H	1.854109	3.236677	2.051208
H	-0.725569	2.551112	2.151364
C	0.561351	2.430094	-0.980516
H	0.777428	1.760935	-1.820426
H	0.214776	3.381264	-1.430733
H	2.711776	2.976673	-0.445346
H	-1.546974	1.898588	-0.263241
O	0.671618	-0.769036	2.150614
O	2.922099	-1.034454	1.775560
O	-1.551422	-0.826287	1.725837
C	1.861447	-0.641043	1.306639
C	-0.429397	-0.538260	1.348530
H	2.119165	-0.055495	-0.797628

TS_*exo*_F_z=+0.008_COSMO(DCM)
E = -2952.00
G = -2884.43
N_{imag} = 1, v = -252.997115

C	0.071006	0.060349	0.071228
C	1.460309	-0.155008	0.095638
C	-0.378023	2.124414	0.003624
H	2.152943	-0.131068	-0.737488
C	1.835007	2.862952	-0.156532
C	1.333461	2.709234	-1.435725
C	-0.023260	2.329235	-1.346540
H	1.883236	2.871462	-2.359305
H	-0.686011	2.169894	-2.195126
C	0.731745	2.721824	0.829902
H	0.992133	2.231015	1.773918

H	0.405053	3.744426	1.108910
H	-0.580051	-0.164395	-0.766917
H	2.851586	3.151919	0.098532
H	-1.411819	2.088723	0.341653
O	0.661837	-0.554241	2.247843
O	2.910885	-0.892548	1.918056
O	-1.560440	-0.514894	1.817554
C	1.857944	-0.532992	1.406026
C	-0.423780	-0.330758	1.423612

Tz_F=0
(BP86/TZ2P//M06-2X/6-31+G(d))
E = -2056.51
N_{imag} = 0

C	0.000000	0.000000	0.000000
C	-0.038892	0.000000	2.505786
N	1.179955	0.000000	0.616349
N	1.159623	0.000000	1.926353
N	-1.218274	0.036912	1.889446
N	-1.197942	0.036912	0.579442
C	-0.062217	0.020912	4.029498
C	0.023974	0.020912	-1.523701
F	0.050346	1.287153	-1.956455
F	1.103200	-0.603599	-1.987982
F	-1.059187	-0.570033	-2.021544
F	-1.160306	-0.570033	4.493485
F	1.002081	-0.603599	4.527048
F	-0.049287	1.287153	4.462862

Ce_F=0
(BP86/TZ2P//M06-2X/6-31+G(d))
E = -1685.34
N_{imag} = 0

C	0.000000	0.000000	0.000000
C	0.000000	0.000000	1.334460
H	-0.887790	-0.042966	-0.624471
H	-0.887805	-0.042959	1.958902
C	1.404110	0.000000	1.895211
C	1.404073	-0.000204	-0.560813
H	1.659699	-0.999383	2.275299
H	1.534267	0.697972	2.729275
H	1.659606	-0.999716	-0.940609
H	1.534366	0.697557	-1.395007
C	2.260961	0.380283	0.667180
H	3.242809	-0.100904	0.667164
H	2.422063	1.463799	0.667052

TS_Tz-CE_F_z=0
(BP86/TZ2P//M06-2X/6-31+G(d))
E = -3733.73
N_{imag} = 1, v = -321.9406

C	1.898887	2.223500	0.000000
C	-0.543345	2.194258	0.000000
C	0.000000	0.000000	0.000000
C	1.379263	-0.000000	-0.098501
H	-0.643060	-0.049992	-0.874046
H	1.895340	-0.003416	-1.053913
C	1.995267	-0.593080	1.154916
H	2.925126	-0.121196	1.477264
H	2.228752	-1.644172	0.931162
C	0.861540	-0.491820	2.190490
H	0.922632	0.464655	2.717396
H	0.906831	-1.286053	2.939224
C	-0.427855	-0.549458	1.350067
H	-1.265818	-0.020390	1.807177
H	-0.748190	-1.589275	1.192418
N	1.315664	2.559277	1.177968
N	0.029086	2.549496	1.178116
N	0.035766	2.523198	-1.192887
N	1.313637	2.540663	-1.192391
C	3.418045	2.241747	-0.021623
C	-2.062089	2.186060	-0.029641
F	-2.572953	1.735756	1.118696
F	-2.515624	1.409179	-1.021280
F	-2.530338	3.421075	-0.232378
F	3.924816	1.937843	1.174866
F	3.899702	1.365928	-0.913592
F	3.864278	3.454188	-0.362491
